# TRAPPC4 regulates the intracellular trafficking of PD-L1 and antitumor immunity

**DOI:** 10.1038/s41467-021-25662-9

**Published:** 2021-09-13

**Authors:** Yimeng Ren, Yun Qian, Luoyan Ai, Yile Xie, Yaqi Gao, Ziyan Zhuang, Jinxian Chen, Ying-Xuan Chen, Jing-Yuan Fang

**Affiliations:** 1 State Key Laboratory for Oncogenes and Related Genes; Division of Gastroenterology and Hepatology, Renji Hospital, School of Medicine, Shanghai Jiao Tong University, Shanghai, China; 2 Department of Medical Oncology, Zhongshan Hospital, Fudan University, Shanghai, China; 3 Division of Gastrointestinal Surgery, Renji Hospital, School of Medicine, Shanghai Jiao Tong University, Shanghai, China

**Keywords:** Cancer immunotherapy, Colorectal cancer, Small GTPases, Transport carrier

## Abstract

Tumor cells evade T cell-mediated immunosurveillance via the interaction between programmed death-1 (PD-1) ligand 1 (PD-L1) on tumor cells and PD-1 on T cells. Strategies disrupting PD-1/PD-L1 have shown clinical benefits in various cancers. However, the limited response rate prompts us to investigate the molecular regulation of PD-L1. Here, we identify trafficking protein particle complex subunit 4 (TRAPPC4), a major player in vesicular trafficking, as a crucial PD-L1 regulator. TRAPPC4 interacts with PD-L1 in recycling endosomes, acting as a scaffold between PD-L1 and RAB11, and promoting RAB11-mediated recycling of PD-L1, thus replenishing its distribution on the tumor cell surface. TRAPPC4 depletion leads to a significant reduction of PD-L1 expression in vivo and in vitro. This reduction in PD-L1 facilitates T cell-mediated cytotoxicity. Overexpression of *Trappc4* sensitizes tumor cells to checkpoint therapy in murine tumor models, suggesting TRAPPC4 as a therapeutic target to enhance anti-tumor immunity.

## Abstract (web summary)

Transport protein particle (TRAPP) is a multimeric protein complex regulating membrane trafficking pathways. Here the authors show that TRAPPC4, a core subunit of TRAPP complex, is required for RAB11-mediated recycling of PD-L1, affecting T-cell-mediated anti-tumor immune responses.

## Introduction

The interaction between T-cell receptors (TCRs) on T cells and peptide-major histocompatibility complexes (MHCs) on target cells comprises the major part of T-cell-based immune elimination of tumors. However, this process is modulated predominantly by a number of co-inhibitory and co-stimulatory ligands and their receptors, known as immune checkpoints^1^. Among these immune checkpoints, the programmed death-1 (PD-1)/PD-1 ligand 1 (PD-L1) axis has emerged as a remarkable therapeutic target in many malignancies. More than 1,000 clinical trials have demonstrated extensive clinical benefits from the immune checkpoint blockade (ICB) therapy targeting the PD-1/PD-L1 axis, which has been approved to treat diverse advanced malignancies including melanoma^2^, non-small cell lung cancer (NSCLC)^3^, microsatellite instable-high (MSI-H) or mismatch repair-deficient (dMMR) colorectal cancer^4^. Although PD-1/PD-L1 blockade has brought about marvelous improvements in patients’ clinical outcomes, only a minority of patients show a durable response to these therapies, and intrinsic resistance remains an intractable challenge. In addition, many patients with common types of cancer, such as non-MSI colorectal cancer, are completely refractory to ICB therapy^5^. Identifying which subset of patients will be responsive to ICB therapy remains a challenge. Previous studies showed that PD-L1 expression on tumor cells, the tumor mutation burden, and T-lymphocyte infiltration might be the key indicators of the  clinical response^6^. PD-L1 expression status appears to be particularly important. In some cancers, such as NSCLC, PD-L1 expression is the most important indicator for ICB therapy, according to The National Comprehensive Cancer Network guidelines. Therefore, sustained efforts should be made to dissect the regulatory process of PD-L1 expression and find combinative therapeutic targets to enhance the efficacy of ICB therapy.

Studies have revealed that both transcriptional and posttranscriptional mechanisms regulate the expression of PD-L1^1,7–9^. Recently, the trafficking and recycling of PD-L1 have been reported to play a crucial role in restoring PD-L1 pools and facilitating tumor immune evasion in the tumor microenvironment (TME)^10–12^. CKLF (chemokine-like factor)-like MARVEL transmembrane domain-containing family member 6 (CMTM6) colocalizes with and maintains PD-L1 at cell membranes and in recycling endosomes, thereby protecting it from lysosomal degradation^10^. Our previous study discovered that Huntingtin interacting protein 1-related (HIP1R) physically interacts with PD-L1 and routes it to lysosomes via the lysosomal targeting signal, thus mediating PD-L1 degradation and altering T-cell-mediated cytotoxicity^11^. These findings suggested that intracellular trafficking might be a potential targetable process to improve the effect of ICB therapy.

Transport protein particle (TRAPP; also known as trafficking protein particle) is a multisubunit protein complex that regulates many membrane trafficking pathways by acting as a canonical tethering component and as guanine nucleotide-exchange factors^13^. Our previous studies revealed that trafficking protein particle subunit 4 (TRAPPC4), a core subunit of TRAPP complex, promotes colorectal carcinogenesis by activating Wnt signaling^14^ and contributes to the aggressiveness of gastric cancer through controlling the transduction of extracellular signal-regulated protein kinase (ERK) signaling^15^. However, whether TRAPPC4 participates in modulating tumor immunity remains unclear. In the present study, murine tumor models show a remarkable CD8^+^ T-cell infiltration in colon tumor areas of intestinal epithelial cell-specific *Trappc4*-deficient (*Trappc4*^△IEC^) mice after azoxymethane (AOM)/dextran sodium sulfate (DSS) challenge. Considering the key role of TRAPPC4 in subcellular membrane trafficking and intestinal cancer progression, we hypothesized that TRAPPC4 participates in regulating PD-L1 cellular trafficking and hence might mediate immune surveillance.

In this work, we find that TRAPPC4 maintains the expression of PD-L1 by acting as a scaffold for PD-L1 and RAB11 to coordinate RAB11-mediated recycling of PD-L1 and protecting PD-L1 from lysosomal degradation, ultimately promoting immune evasion and tumor progression. Loss-of-function screening combined with mass spectrometry analysis indicates that TRAPPC4 is the predominant regulator of PD-L1 expression. These findings reveal the role of the cellular trafficking system in regulating PD-L1 and lead to a deeper understanding of how tumor cells maintain an immune-suppressive state. We also identify TRAPPC4 as a potential therapeutic target, which in combination with ICB, could eliminate immune evasion of tumor cells.

## Results

### TRAPPC4 correlates with the PD-L1 expression in murine and human colorectal cancer tissues

**Fig. 1:**
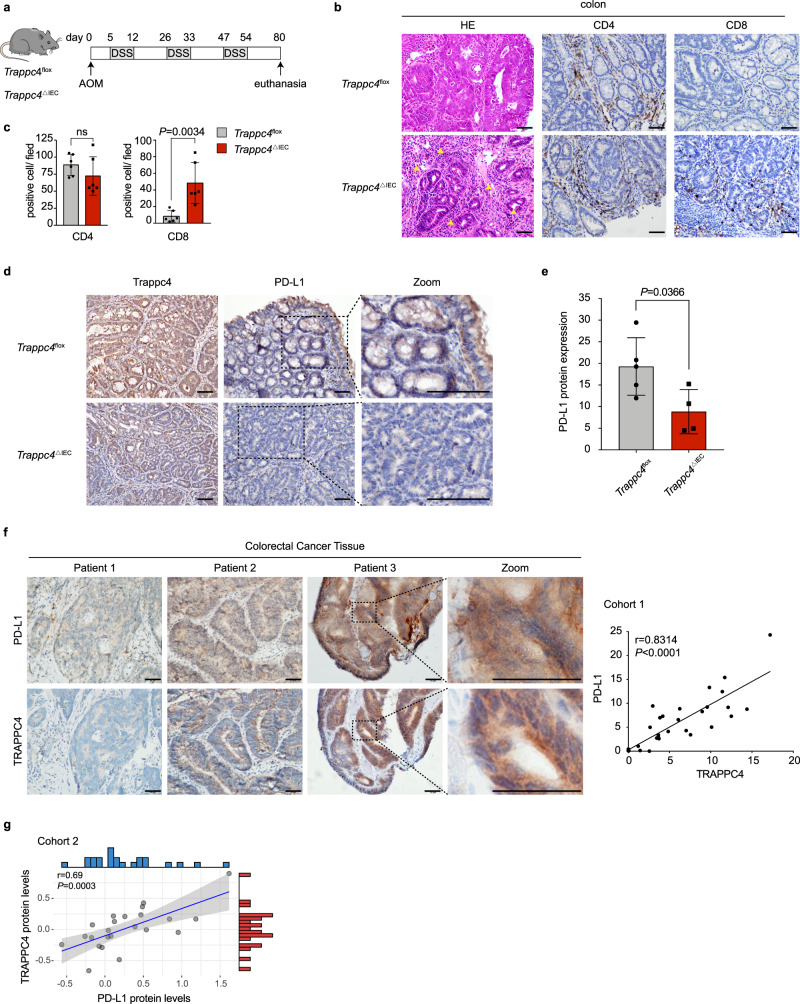
Expression of TRAPPC4 and PD-L1 in animal models and human CRC tissues. **a** Schematic representation of the experimental design and timeline of the mouse models. *Trappc4*^△IEC^ (*n* = 6) and littermate *Trappc4*^flox^ mice (*n* = 6) were challenged with an AOM/DSS CAC induction protocol. Eighty days later, the mice were killed for further analysis. **b**, **c** An enhanced infiltration of CD8^+^ T cells was observed in tumor areas of *Trappc4*^△IEC^ mice. Representative histological images of colon tissues of the mice by hematoxylin and eosin (H&E) staining and immunohistochemical staining of CD4 and CD8. The yellow arrows indicate the infiltration of lymphocytes. Values indicate means ±SD of positive cells from the indicated mice (*n* = 6 per group). Statistical analysis was carried out using a two-tailed Student’s *t* test. ns, not significant. Scale bars, 50 μm. **d**, **e** Immunohistochemical staining of Trappc4 and PD-L1 demonstrated low levels of PD-L1 in the colons of *Trappc4*^△IEC^ mice. The black dotted frames indicate representative fields in the zoomed images. PD-L1 IHC scores were assessed using Image-Pro Plus and values indicate means ± SD from each genotype (*Trappc4*^flox^, *n* *=*  5; *Trappc4*^△IEC^, *n* *=*  4). Statistical analysis was carried out using a two-tailed Student’s *t* test. Scale bars, 50 μm. **f** Immunohistochemical staining showing the clinical relevance between the expression of TRAPPC4 and PD-L1 proteins. Representative images of TRAPPC4 and PD-L1 protein expression in human colorectal cancer tissues using immunohistochemical staining analysis. Correlation between the two proteins was determined by analysis of the Spearman correlation coefficient (*r* value indicated, two-tailed) between TRAPPC4 and PD-L1 protein expression. For each of the 32 samples, five images were taken randomly. TRAPPC4 and PD-L1 IHC scores were assessed by Image-Pro Plus. Scale bars, 50 μm (*P* = 3.8e-9). **g** Correlation of TRAPPC4 and PD-L1 protein expression in colorectal cancer tissues (*n* *=* 23) based on the proteogenomic analysis. Pearson correlation analysis was conducted to determine the correlation of the two proteins (*r* value indicated, two-sided). Error band, 95% confidence band. Source data are provided as a Source Data file.

To investigate whether TRAPPC4 participates in regulating tumor immunity, we first examined the tumor-infiltrating lymphocytes (TILs) and PD-L1 expression in colon tumors with *Trappc4* deficiency. We generated IEC-specific *Trappc4*-deficient (*Trappc4*^△IEC^, heterozygous) mice and exposed them to AOM/ DSS challenge to induce colon tumor formation. Their littermates (*Trappc*4^flox^ mice) were used as controls. Consistent with our previous study^14^, in colitis-associated cancer (CAC) model (Fig. 1a), the *Trappc4*^△IEC^ mice developed significantly fewer tumors in the colon compared with that in their *Trappc4*^flox^ littermates. Remarkably, histological investigations showed salient immune cell infiltration in the tumor areas of the *Trappc4*^△IEC^ mice (Fig. 1b). Further immunohistochemical (IHC) staining showed that the infiltration of CD8^+^ T cells was significantly higher in the tumor areas of *Trappc4*^△IEC^ mice compared with that in *Trappc4*^flox^ mice, whereas no significant difference in CD4^+^ T-cell infiltration was observed (Fig. 1b, c). These observations prompted us to hypothesize that IEC-intrinsic TRAPPC4 regulates tumor immune escape by counteracting CD8^+^ T-cell-mediated cytotoxicity.

In addition to TCR/MHC-mediated recognition, the effect of T-cell-based immune surveillance on tumor cells largely depends on the interaction of a series of immune checkpoints, among which the PD-L1/PD-1 axis acts as a major regulator^1^. Therefore, we detected PD-L1 protein levels in the colon tumors of *Trappc4*^△IEC^ and *Trappc4*^flox^ mice. In line with the increasing TILs in the tumor areas, significantly lower PD-L1 levels were observed in *Trappc4*^△IEC^ mice, indicating that the PD-L1 protein level correlated with that of TRAPPC4 (Fig. 1d, e). Further IHC evaluation of paraffin-embedded human colorectal cancer tissues also showed a strong correlation between the levels of TRAPPC4 and PD-L1 proteins, with *r* = 0.8314 by Spearman’s coefficient analysis (Fig. 1f). Additional proteomic analysis of colorectal cancer demonstrated that tumor TRAPPC4 protein expression positively correlated with that of PD-L1 (Fig. 1g)^16^. Taken together, these results demonstrated that the levels of PD-L1 and TRAPPC4 are correlated in vivo and are clinically relevant in human CRC tissues.

### TRAPPC4 is a predominant regulator of PD-L1 expression

**Fig. 2:**
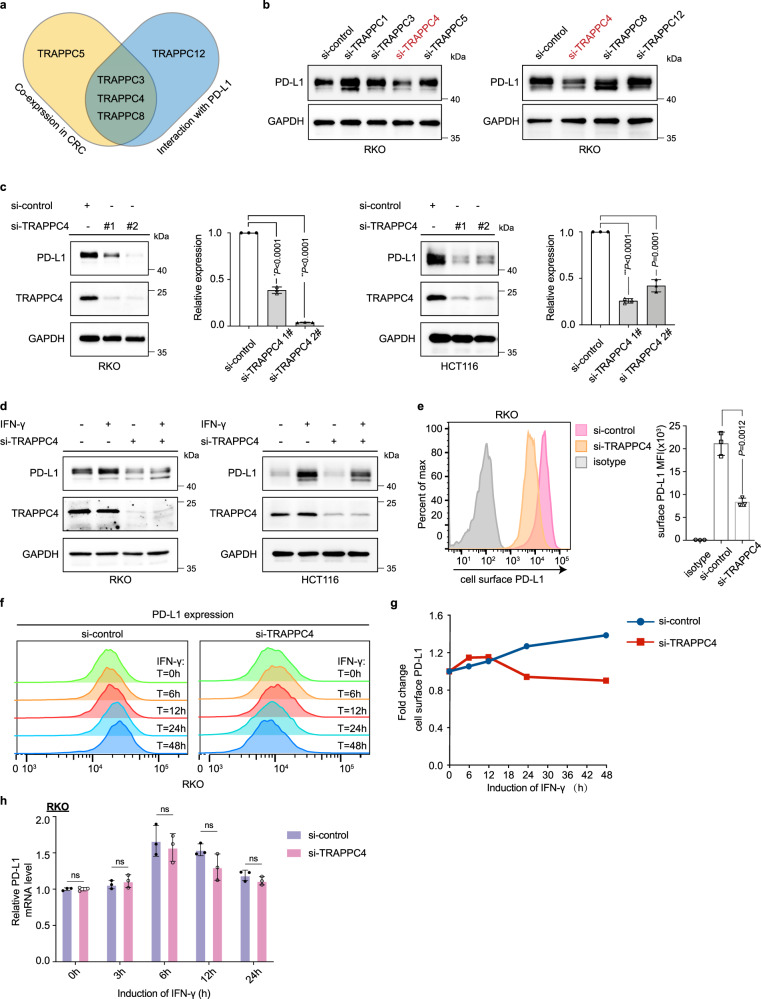
TRAPPC4 is identified as a positive regulator of PD-L1. **a** Venn diagram showing the TRAPP subunits that positively correlated with PD-L1 protein based on the proteogenomic analysis and the TRAPP subunits that interacted with PD-L1 based on the mass spectrometry analysis. **b** Immunoblot showing PD-L1 protein levels in RKO cells transfected with siRNAs targeting different subunits of the TRAPP complexes. **c** Immunoblot for multiple CRC cells treated with control siRNAs or TRAPPC4 siRNAs. GAPDH was used as a loading control. Values indicate means ± SD of the relative gray values of the indicated blots. (*P = 6.9e-6, **P = 3.06e-11, ***P = 7.7e-7). **d** Western blot showing the effect of TRAPPC4 depletion on IFN-γ-induced PD-L1 expression in the indicated cells. **e** PD-L1 expressed on cell surface detected by flow cytometry in RKO cells transfected TRAPPC4-specific siRNA versus the control siRNA. Values indicate means ±SD of the mean fluorescence intensity (MFI). **f**, **g** TRAPPC4 knockdown or control siRNA-transfected RKO cells were induced with IFN-γ at the indicated time points and cell surface PD-L1 was detected using flow cytometry. **h** mRNA levels of PD-L1 in TRAPPC4 knockdown or control RKO cells, with or without the induction of IFN-γ at different time points. Values indicate means ±SD. ns, not significant. Experiments in **b**–**h** were performed three times independently with similar results. Statistical differences were determined using a two-sided Student’s *t* test. ﻿Source data are provided as a Source Data file.

In mammalian cells, there are two well-established TRAPP complexes, TRAPPII and TRAPPIII (Supplementary Fig. 1a). To identify the predominant TRAPP subunit involved in the regulation of PD-L1 expression, we employed a screening approach combining proteomic analysis, mass spectrometry analysis, and a loss-of-function assay. The proteomic analysis of colon cancer indicated that the protein levels of TRAPPC3, TRAPPC4, TRAPPC5, and TRAPPC8 significantly correlated with that of PD-L1^16^ (Fig. 2a, Supplementary Table 1). Further mass spectrometry and co-immunoprecipitation assays identified the interactions between PD-L1 and TRAPPC3, TRAPPC4, TRAPPC8, and TRAPPC12 (Fig. 2a, Supplementary Fig. 1b, c, Supplementary 2). Then, we conducted loss-of-function assays by treating human RKO cells with specific small interfering RNAs (siRNAs) targeting mRNAs encoding the above TRAPP subunits. The knockdown efficiency of the indicated siRNAs was confirmed using quantitative real-time reverse transcription PCR (qRT-PCR; Supplementary Fig. 3a). Loss-of-function screening showed that knockdown of TRAPPC4 diminished the expression of PD-L1 proteins markedly, whereas slight or no reduction of PD-L1 protein was observed upon silencing the expression of other TRAPP subunits (Fig. 2b and Supplementary Fig. 3b, c). To further validate the regulatory role of TRAPPC4 in the expression of PD-L1, we disrupted the expression of TRAPPC4 using two specific siRNAs in different colorectal cancer cell lines, including RKO and HCT-116 cells. Knockdown of TRAPPC4 was confirmed using qRT-PCR (Supplementary Fig. 3d) and western blotting (Fig. 2c). As is shown in the western blot, silencing of TRAPPC4 led to a considerable reduction in the total level of PD-L1 (Fig. 2c) without affecting the level of MHC-I (Supplementary Fig. 3e). Interferon-gamma (IFN-γ) was reported to be the most potent inducer of PD-L1^17^. Therefore, we examined whether knockdown of TRAPPC4 abolished the induction of PD-L1 stimulated by IFN-γ. Following stimulation with IFN-γ, the expression level of PD-L1 was upregulated. However, this induction was largely abrogated upon TRAPPC4 knockdown (Fig. 2d and Supplementary Fig. 3f). Further analysis through flow cytometry indicated that the amount of PD-L1 on the cell membrane was also reduced substantially after the knockdown of TRAPPC4 (Fig. 2e). The increased PD-L1 on cell surface induced by IFN-γ was also attenuated in a time-dependent manner upon silencing of TRAPPC4 (Fig. 2f, g). However, unlike other previously identified regulators of PD-L1^18^, interference of TRAPPC4 expression did not alter the mRNA levels of PD-L1, with or without stimulation by IFN-γ (Fig. 2h), suggesting a mechanism that does not rely on the regulation of mRNA levels. Overall, these results demonstrated that TRAPPC4 regulates PD-L1 levels positively in CRC cell lines.

### The regulation of PD-L1 by TRAPPC4 affects T-cell-mediated antitumor immunity in vitro and in vivo

**Fig. 3:**
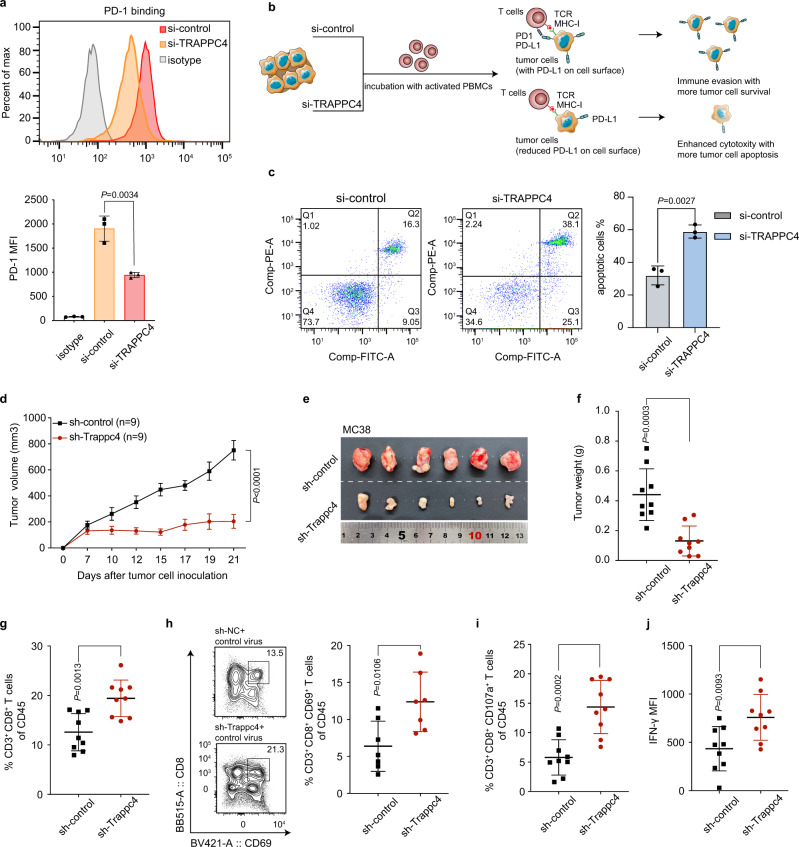
The regulation of TRAPPC4 on PD-L1 affects T-cell-mediated antitumor immunity in vitro and in vivo. **a** Flow cytometry detection of PD-1 bound by cell surface PD-L1 on RKO cells treated with siRNAs targeting TRAPPC4 or control siRNA. The *y* axis indicates the MFI of fluorescence conjugated PD-1. This figure represents the result of three independent experimental replicates. Values indicate means ±SD. **b** Schematic illustration of the experimental procedures for the T-cell-mediated cytotoxicity assay. CRC cells transfected with siRNAs targeting TRAPPC4 or control siRNA were incubated with activated PBMCs. An apoptosis assay was further conducted to determine the T-cell-mediated cytotoxicity. PBMC, peripheral blood mononuclear cell. **c** T-cell cytotoxicity assay against RKO cells treated with a siRNA for TRAPPC4 and control siRNA. Flow cytometry detection of apoptotic cells was conducted after co-incubation of PBMCs with CRC cells. Total apoptotic cells were defined as the proportion of cells in quadrant Q2 and Q3. This experiment was performed three times independently and values indicate means ±SD. Statistical analysis was conducted using a two-sided Student’s *t* test. **d**–**f** Stable MC38 cells transduced with the indicated lentiviruses were injected into *C57BL/6* mice. Statistical analysis of tumor volumes (**d**), representative images of tumors (**e**), and tumor weight (**f**) of mice in different groups (*n* *=* 9 per group) were shown. Values indicate means ±standard error of the mean (SEM) in (**d**) and means ±SD in (**f**), compared by the two-sided Student’s *t* test. (*P* = 1.3e-5 in **d**). **g**–**j** The activation and function of the tumor-infiltrating T cells in each group were analyzed using flow cytometry. The percentages of CD3^+^ CD8^+^ T cells (**g**) (*n* *=* 9 per group), CD3^+^ CD8^+^ CD69^+^ T cells (**h**, the left panels were gated in CD45^+^ subsets) (*n* *=* 7 per group), CD3^+^ CD8^+^ CD107a^+^ T cells of CD45^+^ cells (**i**) (*n* *=* 9 per group) and mean fluorescence intensity (MFI) of IFN-γ of CD8^+^ T cells (**j**) (*n* *=* 9 per group) were plotted as means ±SD compared by the two-sided Student’s *t* test. Source data are provided as a Source Data file.

In the TME, the binding of PD-1 on T cells to PD-L1 on tumor cells compromises T-cell-mediated cytotoxicity toward tumor cells and facilitates an immune-suppressive TME^6^. As knockdown of TRAPPC4 diminished PD-L1 levels and membrane distribution significantly, we set out to assess whether TRAPPC4 affects PD-L1/PD-1 binding and T-cell tolerance. First, we incubated tumor cells with a chimeric protein containing human PD-1 and the Fc fragment of IgG. As shown by flow cytometry, PD-1 binding to tumor cells was dramatically impaired upon silencing of TRAPPC4 (Fig. 3a). In addition, we treated RKO cells with siRNAs targeting other TRAPP subunits to examine their ability to hamper the PD-1 binding of tumor cells (Supplementary Fig. 4a). Intriguingly, only knockdown of TRAPPC4, but not the other tested subunits, reduced the PD-1 binding on the tumor cell surface significantly. Consistently, while silencing of TRAPPC4 induced slight apoptosis of tumor cells (Supplementary Fig. 4b), TRAPPC4-silenced tumor cells were more vulnerable to T-cell cytotoxicity as a result of reduced PD-1/PD-L1 binding. The T-cell-mediated killing assay showed that co-incubation with activated human peripheral blood mononuclear cells (PBMCs) led to significantly more tumor cell apoptosis in the absence of TRAPPC4 (Fig. 3b, c).

To dissect the role of TRAPPC4 in immune checkpoint regulation in vivo, we established stable syngeneic MC38 colorectal cancer cells by transducing lentiviruses expressing short-hairpin RNA (shRNA) to silence Trappc4. We then established subcutaneous tumor models via inoculation of the stable MC38 cells into *C57BL/6* mice. In line with the observation in the *Trappc4*^△IEC^ CAC mouse model, silencing of Trappc4 in MC38 tumor cells eliminated subcutaneous tumor growth significantly (Fig. 3d–f and Supplementary Fig. 5a). IHC staining also confirmed that silencing of mouse Trappc4 in MC38 tumors decreased the level of PD-L1 (Supplementary Fig. 5b), suggesting a regulatory effect of TRAPPC4 on the level of PD-L1 in vivo. Tumor cells exploit the PD-1/PD-L1 axis to inhibit intratumoral immune surveillance, especially blocking the activation and cytotoxicity of T cells^1^. To further delineate the alteration of the TME upon silencing of Trappc4 in tumor cells, we analyzed the TILs in mouse-bearing Trappc4-silenced tumors using flow cytometry assays. Considerable infiltration of CD3^+^ CD8^+^ T cells was detected in sh-Trappc4 expressing tumors compared with the control groups (Fig. 3g and Supplementary Fig. 5c). Then, we employed CD69 as a marker of activated CD8^+^ T cells and CD107a as a degranulation marker^19^ to examine the activation and function of the TILs. Consistently, we detected a significant increase in CD8^+^ CD69^+^ T cells (Fig. 3h) as well as CD8^+^ CD107a^+^ subsets (Fig. 3i and Supplementary Fig. 5d) in live CD45^+^ cells in mouse-bearing sh-Trappc4 expressing tumors. In addition, the levels of IFN-γ in tumor-infiltrating CD8^+^ T cells were elevated in sh-Trappc4 tumors compared with that in the control (Fig. 3j). The above observations suggested that the enhanced T-cell-mediated immunity might be associated with the impaired tumor growth upon silencing of Trappc4.

Taken together, the in vitro and in vivo assays confirmed that TRAPPC4 affected T-cell-mediated antitumor immunity by regulating PD-L1.

### TRAPPC4 interacts with PD-L1 in recycling endosomes

**Fig. 4:**
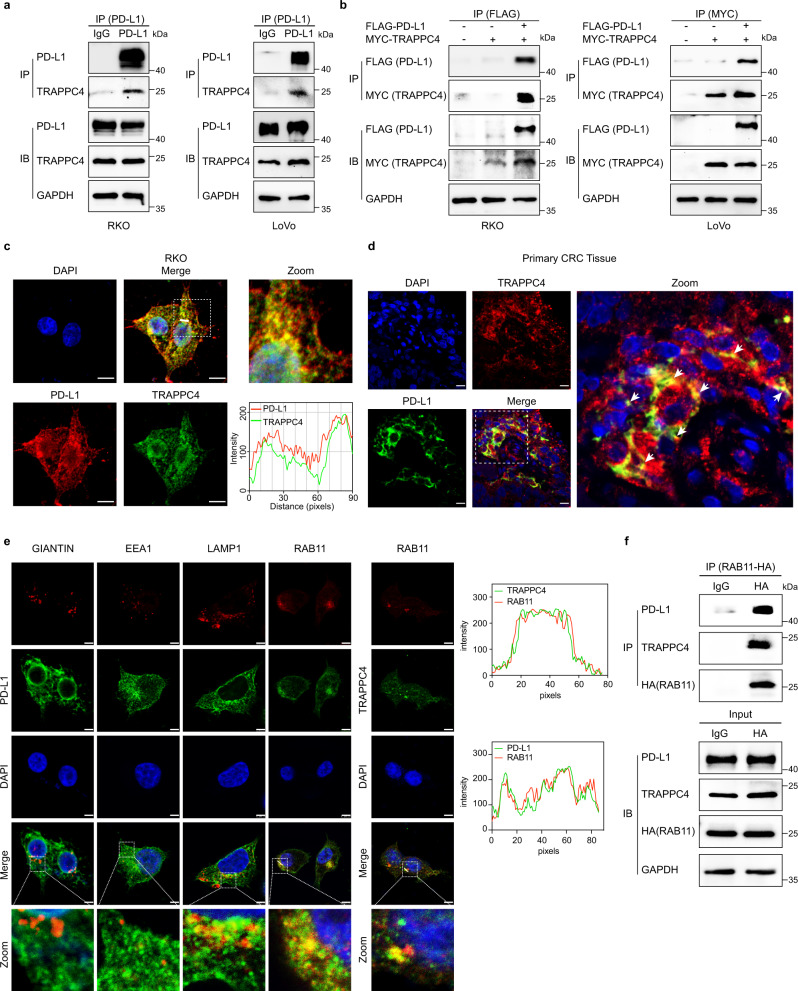
TRAPPC4 interacts with PD-L1 in recycling endosomes. **a** Co-immunoprecipitation assay indicating the interaction between endogenously expressed TRAPPC4 and PD-L1 in RKO (left) and LoVo (right) cells. The control comprised immunoprecipitation with IgG. Lysate (2.5%) was separated as the input. The experiments were repeated three times. **b** RKO (left) and LoVo (right) cells were co-transfected with FLAG-PD-L1 and MYC-TRAPPC4 plasmids and subjected to IP for FLAG and MYC, respectively. Lysate (2.5%) was separated as the input. Experiments were repeated twice. **c** Endogenous PD-L1 and TRAPPC4 mainly colocalize in the plasma of RKO cells. The white dotted frames indicate the representative field shown in the zoomed image. The fluorescence intensity profiles of PD-L1 and TRAPPC4 along the white line are plotted on the right. This figure represents three independent experimental replicates with similar results. Scale bars, 5 µm. **d** Immunofluorescence of TRAPPC4 and PD-L1 in human CRC tissues. The white dotted frame indicates the representative field shown in the zoomed image. White arrows mark the colocalization of PD-L1 and TRAPPC4. This figure represents independent experimental replicates in three human CRC tissues with similar results. Scale bars, 20 µm. **e** Both PD-L1 and TRAPPC4 localized in recycling endosomes. RKO cells were fixed and immunostained for PD-L1 (left panel) or TRAPPC4 (middle panel) together with markers for Golgi (Giantin), early endosome (EEA1), late endosome/lysosome (LAMP1), or recycling endosome (RAB11) before analysis using confocal microscopy. The intensity profiles of RAB11 and PD-L1 as well as RAB11 and TRAPPC4 along the white line were plotted on the right panels. Scale bars, 5 μm. This experiment was repeated three times independently with similar results. **f** Co-immunoprecipitation assays showing the interaction between RAB11 and TRAPPC4 and PD-L1. IgG pulldown was included as a control. Lysate (2.5%) was separated as the input. The experiments were repeated twice independently with similar results. Source data are provided as a Source Data file.

We have demonstrated that interference with TRAPPC4 expression did not alter the mRNA levels of PD-L1, with or without IFN-γ induction, whereas disruption of TRAPPC4 expression dramatically reduced the levels of the PD-L1 protein. Considering that TRAPPC4 is the core subunit of the TRAPPII complex and is a major player in vesicular trafficking^13^, we hypothesized that TRAPPC4 might play a regulatory role in the intracellular trafficking of PD-L1. To verify this hypothesis, we first set out to characterize the interaction between PD-L1 and TRAPPC4. Immunoprecipitation assays showed an interaction between endogenous PD-L1 and TRAPPC4 proteins in multiple CRC cell lines (Fig. 4a). Next, we introduced plasmids expressing FLAG-PD-L1 and MYC-TRAPPC4 into RKO and LoVo cells and confirmed the interaction between these two proteins when expressed ectopically (Fig. 4b). Mass spectrometry assay also validated the interaction of PD-L1 with TRAPPC4 (Supplementary Fig. 1c). In accordance with the above observation, endogenous TRAPPC4 was also found to colocalize with PD-L1 in CRC cell lines (Fig. 4c) and in human CRC tissues (Fig. 4d).

Given that TRAPPC4 is a major tethering factor in the process of intracellular vesicular transportation^13^, we examined whether TRAPPC4 is involved in the vesicular transportation of PD-L1. First, we assessed the subcellular localization of PD-L1 and TRAPPC4. In addition to its expression on the cell surface, PD-L1 was distributed predominantly in lysosomal associated membrane protein 1 (LAMP1)-positive lysosomes and RAB11-positive recycling endosomes (Fig. 4e, left panels). Although our previous study found a colocalization between TRAPPC4 and the Golgi apparatus^15^, we observed that a certain proportion of TRAPPC4 protein was not located in the Golgi. Interestingly, we demonstrated a considerable colocalization between TRAPPC4 and RAB11, suggesting that recycling endosomes are another key organelle for TRAPPC4 vesicles (Fig. 4e, middle panel). Hence, recycling endosomes are probably the subcellular compartments via which TRAPPC4 modulates the trafficking of PD-L1. Indeed, co-immunoprecipitation assays demonstrated a physical interaction between RAB11a and PD-L1 as well as TRAPPC4 (Fig. 4f). Taken together, our results suggested that TRAPPC4 interacts with PD-L1 in RAB11-positive recycling endosomes.

### TRAPPC4 potentially functions as a scaffold for PD-L1 and RAB11 in the process of endosomal recycling of PD-L1 and maintains PD-L1 protein stability

**Fig. 5:**
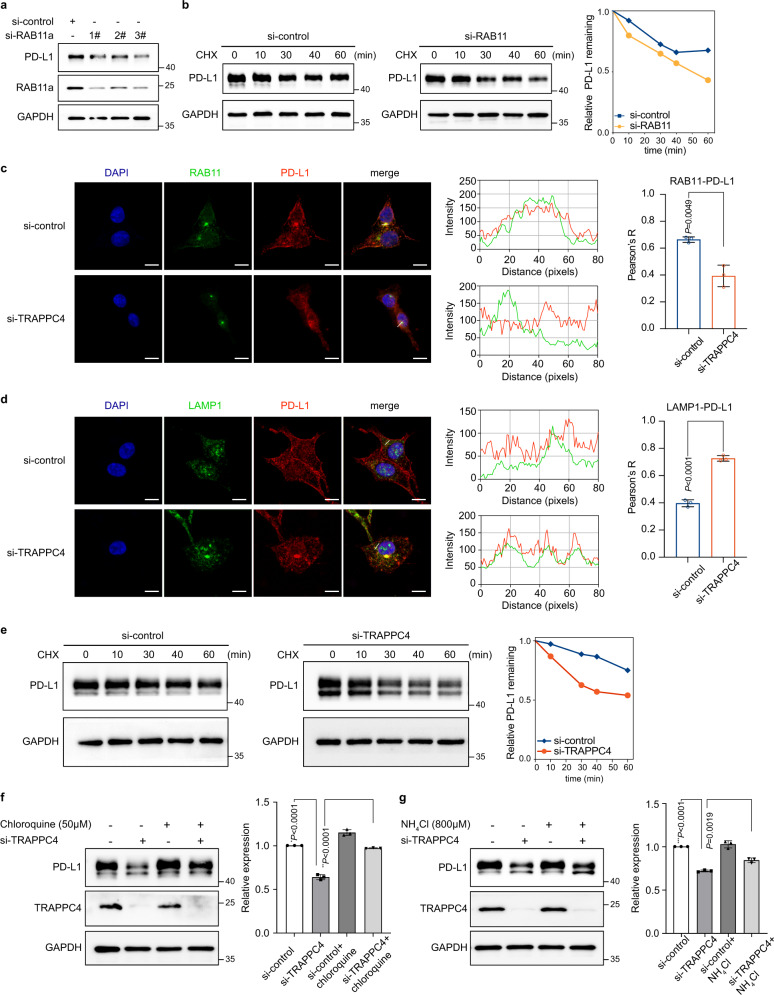
TRAPPC4 maintains PD-L1 expression in recycling endosomes. **a** Disruption of RAB11 downregulated the level of PD-L1 proteins. This experiment was repeated three times independently with similar results. **b** CHX-chase assay indicated an accelerated degradation of PD-L1 proteins upon the knockdown of RAB11 in RKO cells. RKO cells interfered with RAB11 siRNA or control siRNA were incubated with CHX for the indicated time points to monitor the degradation of PD-L1. The degradation speed is quantified on the right. This experiment was repeated three times with similar results. **c**, **d** Immunofluorescence showing the colocalization of PD-L1/RAB11 (**c**) and PD-L1/LAMP1 (**d**) under si-control and si-TRAPPC4 transfection in RKO cells. The intensity profiles of PD-L1/RAB11 and PD-L1/LAMP1 along the white lines were plotted in the middle panel, with the statistical quantification of the colocalizations (Pearson’s *R* value) shown on the right panel. Values are the means ± SD from three independent experiments, compared using two-sided Student’s *t* test. Scale bars, 10 μm. (*P* = 6.26e-5 in **d**) **e** CHX-chase assay indicated accelerated degradation of PD-L1 after disruption of TRAPPC4 expression in RKO cells. RKO cells transfected with siRNA for TRAPPC4 or control siRNA were incubated with CHX for the indicated time points to monitor the degradation of PD-L1. The relative levels of remaining PD-L1 are quantified on the right. This experiment was repeated three times independently with similar results. **f**, **g** RKO cells transfected with TRAPPC4 siRNA or control siRNA were incubated with lysosome inhibitors, chloroquine (50 μM), and NH_4_Cl (800 μM), and analyzed using western blotting. The above experiments were conducted independently three times with similar results. Values are the means ±SD. Statistical differences were determined by two-tailed Student’s *t* test. (*P = 2.75e-5, **P = 4.09e-5, ***P = 1.98e-6). Source data are provided as a Source Data file.

Previous studies have demonstrated that RAB11-positive recycling endosomes act as a restoring pool of PD-L1, supporting its active redistribution to the cell surface^10^. Dynamic transportation of PD-L1 between endocytic recycling compartments and degradation organelles affects the stability and expression of PD-L1 proteins^10,11^. On the assumption that recycling endosomes are the potential subcellular compartment by which TRAPPC4 regulates the level of PD-L1, we first explored the role of RAB11a in sustaining PD-L1 levels. Knockdown of RAB11a using distinct siRNAs significantly reduced the level of PD-L1, providing the direct evidence that recycling endosomes participated in maintaining PD-L1 protein levels (Fig. 5a). Moreover, when we blocked de novo protein synthesis using cycloheximide (CHX), silencing of RAB11a accelerated the degradation of PD-L1(Fig. 5b), suggesting that RAB11a functions in maintaining the level and stability of PD-L1. Sequentially, we set out to investigate how TRAPPC4 affects RAB11-mediated recycling of PD-L1. Immunofluorescence assays indicated abundant localization of PD-L1 in RAB11-marked recycling endosomes in control siRNA-transfected RKO cells, with an intact membrane distribution (Fig. 5c, upper panels). Silencing of TRAPPC4 led to considerable levels of PD-L1 becoming dissociated from recycling endosomes and being rerouted to LAMP1-marked lysosomes (Fig. 5c, d). Accordingly, the distribution of PD-L1 on the cell surface was significantly impaired in the absence of TRAPPC4, which provided additional evidence that knockdown of TRAPPC4 reduced membrane PD-L1 levels (Fig. 2d). In addition, the co-immunoprecipitation assay demonstrated that silencing of TRAPPC4 reduced the interaction between PD-L1 and RAB11a (Supplementary Fig. 6a). Notably, the knockdown of TRAPPC4 did not alter the level of RAB11a (Supplementary Fig. 6b). These results indicated that TRAPPC4 potentially acts as a scaffold protein for RAB11 and PD-L1 in coordination with the endosomal recycling of PD-L1.

Consistent with the observation that an increased proportion of PD-L1 colocalized with lysosomes upon TRAPPC4 silencing, a CHX-chase assay demonstrated accelerated degradation of PD-L1 in the absence of TRAPPC4 (Fig. 5e). In addition, incubation with lysosome selective inhibitors, chloroquine and NH_4_Cl, both alleviated the degradation of PD-L1 after TRAPPC4 knockdown (Fig. 5f, g). In addition, we performed CHX-chase assays in cells silenced for the expression of other subunits of the TRAPPII and TRAPPIII complexes and observed no significant alteration of PD-L1 stability (Supplementary Fig. 6c). These results indicated that lysosomal degradation was at least partly involved in PD-L1 degradation after TRAPPC4 knockdown. In summary, TRAPPC4, as a major tethering factor, coordinates the localization of PD-L1 in recycling endosomes and sustains the stability of PD-L1 by protecting it from lysosomal degradation, thus facilitating endosomal recycling of PD-L1.

### Overexpression of Trappc4 augments the efficacy of checkpoint blockade therapy

**Fig. 6:**
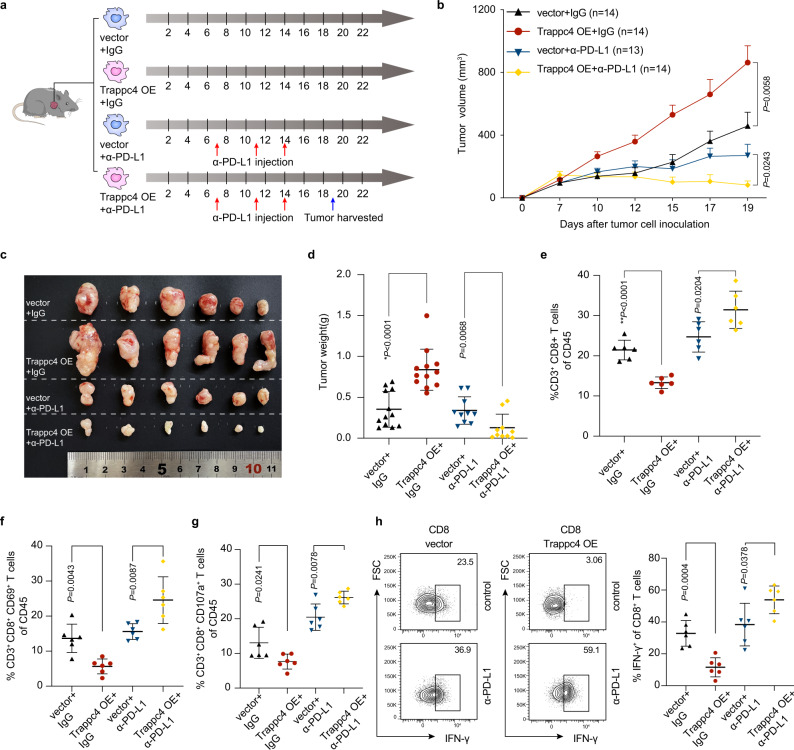
Overexpression of Trappc4 affects tumor microenvironment composition and immunotherapy efficacy. **a** Schematic representation of the xenograft mouse model and dosage regimen. Mice bearing control or Trappc4-OE MC38 tumors were treated with anti-PD-L1 mAb. **b**–**d** Statistical analysis of tumor volumes (**b**) (vector+α-PD-L1, *n* = 13; all other groups *n* = 14), representative images of tumors (**c**) and tumor weights (**d**) (vector + IgG, *n* = 12, Trappc4-OE + IgG, *n* = 12, Vector+α-PD-L1, *n* = 10, Trappc4-OE + α-PD-L1, *n* = 10) of the mice with subcutaneous tumors in different groups. Values indicate means ±SEM in (**b**) and means ±SD in (**d**), compared by nonparametric Mann–Whitney test, two-sided. (^*^P = 7.2e-5) **e**–**h** The percentages of CD8^+^ T cells (**e**), CD69^+^ CD8^+^ T cells (**f**), CD107a^+^ CD8^+^ T cells of CD45^+^ cells (**g**) and IFN-γ^+^ cells of CD8^+^ T cells (**h**) in each group (*n* = 6 per group) were analyzed by flow cytometry. The statistics were plotted as means ±SD, compared by two-sided Student’s *t* test in **e**, **g**, **h**, and nonparametric Mann–Whitney test in **f**. OE, overexpression. (^**^P = 3.6e-5). Source data are provided as a Source Data file.

In mouse-bearing sh-Trappc4-expressing MC38 tumors, we observed an increased infiltration of activated CD8^+^ T cells as well as an enhanced antitumor immune microenvironment., suggesting a regulatory role of TRAPPC4 in tumor immunity. We then speculated whether TRAPPC4 would affect the efficacy of checkpoint blockade therapy, which is of great importance for the introduction of TRAPPC4 as a potential therapeutic target to augment checkpoint therapy. In this regard, we generated stable Trappc4-overexpressing (OE) MC38 cells, and inoculated Trappc4-OE cells and control cells into *C57BL/6* mice, with or without the treatment of mouse anti-PD-L1 monoclonal antibody (mAb). A schematic representation of the model is depicted in Fig. 6a. Trappc4 expression in the stably transfected cells was validated using IHC staining (Supplementary Fig. 7a). As expected, overexpression of Trappc4 dramatically promoted the growth of tumors in the mice (Fig. 6b–d and Supplementary Fig. 7b). Remarkably, Trappc4-OE MC38 tumors exhibited a better response to the anti-PD-L1 mAb, with impaired tumor growth, smaller tumor volumes, and reduced tumor weights. (Fig. 6b–d and Supplementary 7b). This interesting observation demonstrated that Trappc4 sensitized MC38 tumor cells to respond to anti-PD-L1 therapy. We further characterized the alteration of the TME in Trappc4-OE mouse tumors in response to checkpoint blockade treatment. In line with the previous observations in the *Trappc4*^△IEC^ CAC models and sh-Trappc4 expressing MC38 tumor models, overexpression of Trappc4 inhibited CD8^+^ T-cell-mediated antitumor immunity, with less infiltration of CD8^+^ T cells in tumor areas (Fig. 6e and Supplementary Fig. 8a), reduced T-cell activation marked by CD69^+^ CD8^+^ T-cell subsets (Fig. 6f and Supplementary Fig. 8b) and fewer degranulation populations marked by CD107a (Fig. 6g and Supplementary Fig. 8c). A decrease in the number of IFN-γ^+^ CD8^+^ T cells also indicated fewer cytotoxic T cells in Trappc4-OE tumors (Fig. 6h). By contrast, flow cytometry analysis showed that Trappc4 overexpression in MC38 cells drove enhanced antitumor immunity in response to anti-PD-L1 therapy, including pronounced CD8^+^ T-cell infiltration, and increased proportions of CD69^+^ CD8^+^ T cells and CD107a^+^ CD8^+^ T cells (Fig. 6e–g and Supplementary Fig. 8a–c). The proportion of IFN-γ-producing CD8^+^ T cells also increased in anti-PD-L1 treated Trappc4-OE tumors (Fig. 6h). These results indicated that overexpression of Trappc4 enhanced the effects of anti-PD-L1 therapy in a preclinical mouse model.

## Discussion

**Fig. 7:**
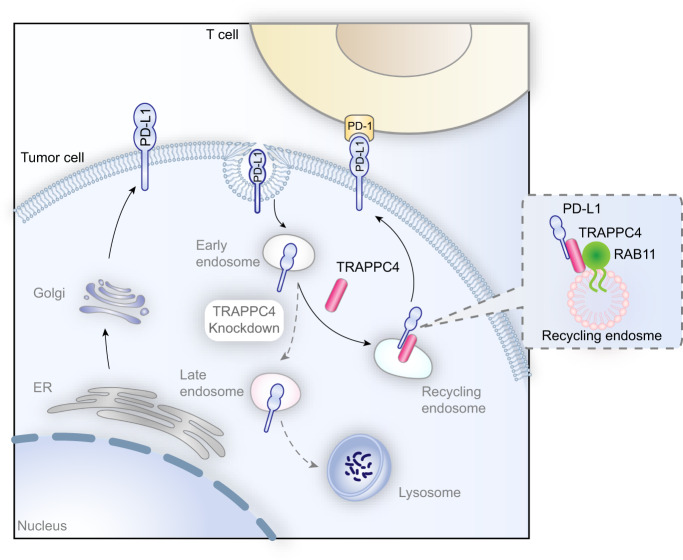
TRAPPC4 maintains the expression of PD-L1 by regulating its intracellular trafficking. In the tumor cell cytoplasm, TRAPPC4 acts as a scaffold for endocytosed PD-L1 and RAB11, thus facilitating the RAB11-mediated recycling of PD-L1 and restoring the distribution of PD-L1 on the cell surface. Disruption of TRAPPC4 in CRC cells leads to a redistribution of endocytosed PD-L1 to lysosomes and its degradation, resulting in enhanced T-cell-mediated cytotoxicity toward colorectal tumor cells.

Antitumor immunotherapy targeting the PD-1/PD-L1 checkpoint has provided substantial clinical benefits in multiple malignancies in recent years. However, the small proportion of durable remission and non-negligible intrinsic resistance hinders the more extensive application of ICB in the majority of cancer types. One of the most common malignancies that are refractory to ICB is mismatch repair proficient colorectal cancer, which accounts for ~85% of cases of colorectal cancer^4^. The MSI state has been reported as the main predictor of the clinical response of patients with colorectal cancer. The overall response rate of patients with MSI-H/dMMR colorectal cancer treated with PD-1 blockade was no >50% in a clinical trial^20^, indicating the complex immunosuppressive mechanisms in colorectal cancer. Previous efforts have been made to block PD-L1 on tumor cells. However, the intracellular storage and redistribution of endocytosed PD-L1 to cell surface diminish the efficacy of ICB^10^. In this study, we focused on the intracellular transportation of PD-L1 and showed that TRAPPC4 functions as a vesicular trafficking protein facilitating the endosomal recycling of PD-L1 to the cell membranes (Fig. 7). TRAPPC4 interacts with PD-L1 and RAB11-marked recycling endosomes, where it acts as a scaffold for PD-L1 and RAB11, and protects PD-L1 from being targeted for lysosomal degradation. Silencing of TRAPPC4 led to the sequential degradation of PD-L1 in lysosomes, thus reducing the overall PD-L1 reserve in tumor cells, as well as that on the cell surface. The specific depletion of TRAPPC4 in tumor cells enhanced T-cell-mediated cytotoxicity toward tumor cells in vitro and augmented antitumor immunity in vivo. By contrast, overexpression of Trappc4 made MC38 tumors more sensitive to checkpoint blockade therapy. This observation is possibly due to the positive regulation of TRAPPC4 on the level of PD-L1. The upregulation of PD-L1 levels in TRAPPC4-OE tumor cells (Supplementary Fig. 7a) led to a suppressive TME with less infiltration of cytotoxic CD8^+^ T cells, which may be one of the predominant reasons accounting for the overgrowth of TRAPPC4-OE tumors. Thus, treatment with PD-L1 blockade in Trappc4-OE tumors achieved better efficacy. In summary, the results of the present study identified TRAPPC4 as a previously uncharacterized regulator of PD-L1 in subcellular trafficking and suggested it as a potential therapeutic target to enhance immune checkpoint therapy.

Our previous studies^14,15^ showed that the expression of TRAPPC4 is associated positively with the clinical features of gastrointestinal malignancies including tumor volume, lymph node invasion, distant metastasis, tumor-node-metastasis staging, and overall survival of patients. The present study extended the research to depict the role of TRAPPC4 in regulating PD-L1 and T-cell-mediated antitumor immunity. In mammalian cells, TRAPPC4 is a subunit of the TRAPPII and III complexes, mediating the vesicle tethering process. Multiple screening assays including proteomic analysis, LC-MS/MS analysis, and the loss-of-function test indicated that TRAPPC4 is a predominant regulator of PD-L1, and a significantly positive correlation between the protein levels of TRAPPC4 and PD-L1 was demonstrated in murine and human colon cancer tissues. Using in vivo and in vitro loss-of-function assays, we identified TRAPPC4 as a major player in regulating tumor immunity. Further detailed studies focused on the role of other TRAPP subunits in the subcellular trafficking process of PD-L1 are needed. Taken together, our data revealed TRAPPC4 as a key TRAPP component required for maintaining PD-L1 protein levels and stability in CRC cells.

Cellular transportation of proteins via exocytic and endocytic pathways relies on the elaborate coordination of vesicular-tubular transport. A typical membrane trafficking procedure involves four steps: vesicle formation, subcellular transport, vesicle tethering, and membrane fusion^21^. The specificity of membrane tethering and fusion is critical for proper and correct vesicular transportation, which is mediated by Rab GTPase^21^. In line with the previous studies^10^, we observed that RAB11 mediates the recycling process of endocytosed PD-L1. We further concluded that the knockdown of RAB11 accelerated the degradation of PD-L1. As another canonical tethering factor, TRAPPC4 interacted with PD-L1 as well as RAB11 to facilitate the RAB11-mediated recycling of PD-L1. Our findings suggested that there might be potential coordination of tethering factors in transporting and sustaining the stability of PD-L1.

Previously, TRAPPC4 was identified as a molecular scaffold controlling the Ras-Raf-MEK-ERK signaling pathway^15^. Furthermore, its encoding gene is a direct transcriptional target of nuclear factor-κB^15^. Considering that aberrant activation of the above oncogenic signaling pathways is also involved in the regulation of PD-L1^22–25^, further investigation is warranted as to whether TRAPPC4 has additional roles in the regulation of PD-L1, in which it might interact with other signaling molecules and may act as a molecular adapter between oncogenic signals and PD-L1.

In summary, our study revealed the subcellular trafficking of PD-L1. We showed that TRAPPC4, a canonical vesicular tethering factor, functions together with RAB11 to route endocytosed PD-L1 to recycling compartments, thus replenishing PD-L1 in the cell membrane and promoting immune evasion. The key role of TRAPPC4 in the regulation of PD-L1 adds another piece of crucial information concerning the spatial transportation of PD-L1. The finding that Trappc4-OE MC38 tumors were more sensitive to ICB makes a potential case for TRAPPC4 as a therapeutic target and predictor of the response to ICB.

## Methods

### Antibodies and reagents

The antibodies and reagents used in this study were described in detail in Supplementary Table 2.

### Patient specimens

Human tissue specimens were collected from 32 patients with CRC from Renji Hospital affiliated with Shanghai Jiao Tong University School of Medicine (Shanghai, China). Clinical characteristics of the patient were shown in Supplementary Table 3. Mucosal biopsies were taken from colons of patients with colorectal tumors. The greatest dimension of the biopsies was at least 0.5 cm and histologically confirmed CRC tissues were collected for further examinations. The study was conducted with approval from the ethics committee of Renji Hospital, Shanghai Jiao Tong University School of Medicine (Shanghai, China) and written informed consent was obtained from all enrolled patients. All the research was carried out under the guidance of the provisions of the Declaration of Helsinki of 1975.

### Animal experiments

The IEC-specific *Trappc4*-deficient mice were constructed using a knockout (KO)-first strategy^26^ in the *C57BL/6* background^14^. The genotype was identified using PCR analysis and Southern blotting. Eight to ten-week-old male *Trappc4*^△IEC^ mice and their littermates *Trappc4*^flox^ mice were then exposed to a CAC challenge based on an AOM/DSS regimen^27^. In brief, mice were injected with AOM at 10 mg/kg body weight intraperitoneally. Five days later, mice were fed with 2.5% DSS in drinking water for 1 week and sequentially with fresh, regular drinking water for 2 weeks. The drinking cycle of DSS was repeated three times and the mice were killed for analysis four weeks after the last DSS administration. The colons and rectums of the mice were cut longitudinally and flushed with phosphate-buffered saline (PBS). Tumors and lesions were examined carefully and recorded. Neutral buffered formalin (10%) was used to fix the distal part of the colons for 24 h. After dehydration with ethanol, the colon tissues were embedded in paraffin for further histological analysis.

To establish subcutaneous tumor models, 6–8-week-old male *C57BL/6* mice were purchased from Shanghai Model Organism Center (Shanghai, China) and quarantined for 1 week before inoculation of MC38 cells (mouse colon adenocarcinoma cells). Stable MC38 cells (8 × 10^5^) were suspended in 100 μl of PBS and injected subcutaneously into the flanks of the mice. The mice tumor volumes were measured every 2–3 days. Tumor volume (mm^3^) was calculated as π/6 × length × width^2^. For treatment with checkpoint therapy, 200 μg of an anti-PD-L1 monoclonal antibody (BE0101; BioXcell, Lebanon, NH, USA) or isotype IgG were injected intraperitoneally into each mouse on day 7, 11 and 14. The mice were killed humanely at the indicated time point after tumor cells inoculation and tumors were harvested for subsequent analysis. All mice were maintained under specific-pathogen-free housing with a maximum of five mice per cage. The mice were maintained at 25°C, with suitable humidity (typically 50%) and a 12-hour dark/light cycle. All animal experiments were conducted according to the guidelines approved by the Institutional Animal Care and Use Committee of Renji Hospital, Shanghai Jiaotong University School of Medicine.

### Construction of *Trappc4*^△IEC^ mouse

The *Trappc4*-null mouse line was constructed based on the knockout (KO)-first strategy in the *C57BL/6* background^14^. Through this strategy, a cassette was inserted into the third exon of the *Trappc4* gene, which produced a knockout phenotype at the transcript level as a result of the presence of a splice acceptor in the cassette capturing the transcript. Thus, mice homozygous for the KO-first cassette was *Trappc4*-null. Conditional KO mice can be generated by crossing *Trappc4* KO-first mice with flippase deleter (Flper) mice, which induced excision of the cassette at the FRT site1. This crossing step restored gene function while made *Trappc4* exon3 flanked by two loxP sites. Using appropriate Cre-expressing mouse lines then allowed for cell-type-specific *Trappc4* gene deletion. Here, we used a Villin-Cre mouse line to specifically delete the *Trappc4* gene in enterocytes.

### Tumor sample digestion and flow cytometry analysis

The subcutaneous tumors were excised, cut into small pieces, and digested with collagenase D (1 mg/ml, Roche) and DNase I (0.2 mg/ml, Roche) at 37°C for 30 min. After digestion, the samples were passed through 70 μm cell strainers to make a single-cell suspension, followed by a staining procedure with fluorescently-labeled antibodies. For cytokine staining, the cells were incubated in a culture medium containing Leukocyte Activation Cocktail (BD Biosciences) at 37°C for 4 h. The cells were stained with anti-CD45 (BD Biosciences, 557659), anti-CD3 (eBioscience, 45-0031-82), anti-CD8 (Biolegend, 100706), anti-CD69 (Biolegend, 104528) and anti-CD107a (Biolegend, 121614). After the extracellular staining procedure, the cells were washed and resuspended in Fixation/Permeabilization solution (BD Biosciences), followed by staining with anti-IFN-γ (BD Biosciences, 557649). Samples were analyzed quantitatively by fluorescence-activated cell sorting (FACS) (BD Biosciences, San Jose, CA, USA). All staining steps were protected from light. Statistical analysis was performed using FlowJo (BD Biosciences). The detailed antibody information is included in Supplementary Table 2. The gating strategy is shown in Supplementary Fig. 9a.

### IHC and immunofluorescence

For IHC, paraffin-embedded colon tissues were cut into four-micrometer thick sections. After rehydration, heat-induced antigen retrieval was performed using citrate buffer (MXB biotechnologies, Fuzhou, China; MVS-0066) or EDTA (MXB biotechnologies; MVS-0099) solution according to the manufactures’ instructions of the primary antibodies. After endogenous peroxidases blocking and blockade with normal serum, the sections were incubated with primary antibodies at 4°C overnight and with secondary antibodies at room temperature for 30 min. IHC staining was detected using an ABC Kit Vectastain Elite (Vector Laboratories, Burlingame, CA, USA) and diaminobenzidine substrate (DAKO and Vector Laboratories). Protein expression was assessed on the basis of the intensity and the scale of staining as described previously^28^ using Image-Pro Plus 6.0 (Media Cybernetics, Rockville, MD, USA).

For immunofluorescence, RKO cells were plated in eight-well chamber slides (154534, Thermo Scientific, Waltham, MA, USA) at a confluence of ~50%. Cells were then fixed with 4% paraformaldehyde buffer for 20 min and washed with PBS three times (5 min each time). After permeabilizing with 0.2% Triton-X-100 (Sigma-Aldrich, St. Louis, MO, USA) for 10 min and blocking in 1% bovine serum albumin (BSA) in PBS for 1 h at room temperature, the cells were incubated with primary antibodies at 4°C overnight, and then with fluorescently-labeled conjugated secondary antibodies (Alexa Fluor 594 or 488; 1:500; Invitrogen, Waltham, MA, USA) at room temperature for 30 min. After washing with PBS three times, the slides were stained with 2-(4-amidinophenyl)-1H-indole-6-carboxamidine (DAPI) and sealed using coverslips. The slides were examined under a Zeiss LSM710 confocal microscope (Carl Zeiss, Oberkochen, Germany) equipped with a ×40 oil immersion objective. The confocal microscopy software of the ZEN system 2011 Black Edition was used to capture images. Colocalization analysis was performed using the colocalization plugin of ImageJ (2.1.0) software (NIH, Bethesda, MD, USA). Antibodies used in IHC and immunofluorescence are listed in Supplementary Table 2.

### Cell lines and transfection

Human colorectal cancer cell lines HCT-116, RKO, and LoVo were purchased from the ATCC (Manassas, VA, USA) and mouse colorectal cancer cell line MC38 were purchased from BMCR. All these cell lines were cultured at 37°C in a humidified incubator with 5% CO_2_. HCT-116 Cells were maintained in McCoy’s 5 A medium (Gibco, Grand Island, NY, USA) supplemented with 10% FBS (fetal bovine serum; Invitrogen). RKO cells and LoVo cells were maintained in Roswell Park Memorial Institute 1640 medium (Gibco) supplemented with 10% FBS (Invitrogen). MC38 cells were maintained in Dulbecco’s Modified Eagle Medium supplemented with 10% FBS. For transfection of short interfering RNAs (siRNAs), cells were seeded at 60% confluence in six-well plates at 24 h before transfection. Five microliters of siRNA for a specific target and 5 μl of DharmaFECT1 Transfection Reagent (GE Healthcare, Chalfont St Giles, UK) were added to 195 μl Opti-MEM (11058021, Gibco) separately and left at room temperature. After 5 min, the above two parts of the transfection complex were mixed by pipetting, incubated for 15 min at room temperature, and added into the well. About 24–48 h after the transfection, the cells were harvested for further analysis. For transfection of plasmid, we used the FuGENE Transfection HD Reagent (Promega, Madison, WI, USA). Cells were harvested ~48 h after transfection for further analysis. ﻿The sequences of siRNAs used for transfection are shown in Supplementary Table 4.

### Western blotting and immunoprecipitation

Western blotting analysis was performed using standard techniques. Cells were collected and lysed using radioimmunoprecipitation assay (RIPA) buffer supplemented with a protease inhibitor cocktail. A bicinchoninic acid (BCA) protein assay kit (Thermo Fisher) was used to determine the concentration of the protein in the samples. Protein samples (25–30 µg) were separated using 10% sodium dodecyl sulfate-polyacrylamide gel electrophoresis (SDS–PAGE) before being transferred to a polyvinylidene fluoride membrane (Bio-Rad, Hercules, CA, USA). The membrane was blocked with 5% fat-free milk in Tris-buffered saline buffer for 1 h at room temperature and then incubated with the respective primary antibodies overnight at 4°C. Horseradish peroxidase-conjugated secondary antibodies (1: 5000 dilution) were incubated for 1 h at room temperature. Specific bands were developed using an ECL Kit (32106, Thermo Fisher). Immunoreactive protein bands on the western blots were quantified using the ImageJ software.

Immunoprecipitation (IP) was performed as described previously^11^. After transfection with the indicated plasmids for 48 h, cells were lysed using IP lysis buffer (Thermo Fisher) containing a proteinase and phosphatase inhibitor cocktail. A 2.5% aliquot of the lysate from each sample was collected to indicate the number of input proteins. Two microliters of the indicated antibody were added to each sample and the mixture was rotated at 4°C overnight. In parallel, 20 μL of Protein G Agarose beads (Thermo Fisher) for each sample were blocked in 1% BSA with rotation overnight at 4°C. On the next day, the beads were removed from the blocking solution using centrifugation, followed by the addition of the mixture of cell lysate with the indicated antibody and rotated at room temperature for 1 h. Then the lysate was decanted and the beads were washed with 500 μL of PBS three times by rotation and then boiled for further analysis. Both input and immunoprecipitated samples were separated using SDS–PAGE electrophoresis. Detailed information on antibodies is shown in Supplementary Table 2.

### QRT-PCR

Total cell RNA was extracted with RNAiso Plus (Takara). For each sample, 1 μg of total RNA was reverse transcribed to cDNA using the PrimeScript™ RT Master Mix (Takara). Quantitative real-time PCR was then conducted in triplicates using the TB Green PCR Master Mix reagent as the detector on an Applied Biosystems 7900 quantitative PCR system (Applied Biosystems, Foster City, CA, USA), as previously described^29^. The target transcript level was normalized to the expression of glyceraldehyde-3-phosphate dehydrogenase mRNA. The 2^−ΔΔCT^ method was used to quantify the relative expression levels, which were indicated as the fold-change. The sequences of primers used for qPCR assays are shown in Supplementary Table 5.

### Detection of PD-L1 on the cell surface

After transfection with siRNA or induction with IFN-γ for the indicated time, cells were trypsinized and collected into different centrifuge tubes and washed with PBS twice. The cells were then incubated with allophycocyanin (APC)-conjugated anti-human PD-L1 antibody (1:250) (BioLegend, San Diego, CA, USA) for 30 min on ice. After incubation, the stained cells were washed twice with cold PBS and analyzed by FACS. All staining steps were protected from light. The gating strategy used for flow cytometry is shown in Supplementary Fig. 9b.

### PD-L1/PD-1-binding assay

At 48 h after transfection with siRNA, cells were trypsinized and collected into centrifuge tubes. After incubation with recombinant human PD-1 Fc chimeric protein (R&D Systems, Minneapolis, MN, USA) at room temperature for 30 min, the cells were washed and with staining buffer twice, followed by incubation with Alexa Fluor 488 dye conjugated anti-human antibodies (1:500, Thermo Fisher) at room temperature for 30 min. After resuspension in staining buffer, samples were analyzed quantitatively by flow cytometry. All staining steps were protected from light. Statistical analysis was performed using FlowJo (BD Biosciences).

### T-cell cytotoxicity assay

To obtain activated T cells, human PBMCs (StemEry Biotech, Fuzhou, China) were treated with a T-Cell Activation/Expansion Kit (130-091-441, Miltenyi Biotec, Bergisch Gladbach, Germany) for 24 h following the manufacturer’s instruction. At the same time, RKO cells treated with the TRAPPC4 siRNA or control siRNA were seeded into a 96-well plate at a density of 3000 cells per well. Then, RKO cells were then co-cultured with activated PBMC cells at a ratio of 1:10 in the 37°C incubators for 6 h. To detect apoptotic cells, all the cells were harvested with trypsin into different centrifuge tubes and incubated with APC-conjugated anti-human CD45 antibodies (BD Biosciences) in staining buffer at 4°C for 30 min in the dark. The cells were then washed twice with cold PBS and stained with Annexin V fluorescein isothiocyanate/propidium iodide (BD Biosciences) following the manufacturer’s protocol to detect apoptotic cells. Apoptosis of the CD45^−^ cells (tumor cells) was further examined using flow cytometry.

### Mass spectrometry

The RKO cells were lysed using IP lysis buffer (Thermo Fisher) containing a proteinase and phosphatase inhibitor cocktail. Then the lysis was incubated and precipitated with the anti-PD-L1 antibody, and the precipitation of IgG was set as a negative control. The above-immunoprecipitated samples were separated using SDS–PAGE electrophoresis, stained with Coomassie, and cut into ~1 mm square pieces. Gel pieces were destained in 50 mM NH_4_HCO_3_ in 50% acetonitrile (v/v) until clear, washed three times in ddH_2_O, and dehydrated with 100 μl of 100% acetonitrile for 5 min. Then the gel pieces were rehydrated in 10 mM dithiothreitol and incubated at 25°C for 60 min. The gel pieces were washed with NH_4_HCO_3_ buffer once and incubated with 55 mM iodoacetamide at room temperature for 45 min, protecting from the light. Gel pieces were washed with 50 mM NH_4_HCO_3_ twice and dehydrated with 100% acetonitrile. Then the gel pieces were digested using 10 ng/μl Trypsin at 37°C for over 16 h. Peptides were extracted with 50% acetonitrile/5% formic acid twice, followed by dehydration using 100% acetonitrile. The samples were freeze-dried to completion, dissolved in 0.1% formic acid ﻿and desalted with C18 Zip Tips, and dried with SpeedVac. The samples were resuspended with 0.1% formic acid for mass spectrometry analysis.

In brief, samples were first loaded onto an HPLC chromatography system named Thermo Fisher Easy-nLC 1000 equipped with a C18 column (1.8 mm, 0.15 × 1,00 mm). Solvent A contained 0.1% formic acid and solvent B contained 100% acetonitrile. The elution gradient was from 4% to 25% solvent B in 80 min, 25% to 35% in 4 min and climbing to 90% in 2 min, then holding at 90% for 8 min, all at a constant flow rate of 300 nl/min. The peptide samples were then analyzed on Thermo Fisher LTQ Obitrap ETD mass spectrometry in the positive-ion mode with an automated data-dependent MS/MS analysis with full scans (350–1600 m/z) acquired using FTMS at a mass resolution of 30,000. The MS/MS was acquired using higher-energy collision dissociation at 35% collision energy at a mass resolution of 15,000. The mass spectrometry analysis was carried out at the AIMSMASS Co., Ltd. (Shanghai, China).

All Raw files were searched by Proteome Discoverer 1.4 (Thermo Fisher Scientific) against the Uniprot-Homo sapiens. fasta. Trypsin was specified as a cleavage enzyme allowing up to two missing cleavages. A mass error was set to 20 ppm for precursor ions and 0.05 Da for fragment ions. ﻿The search included variable modifications of methionine oxidation and deamidation, and fixed modification of carbamidomethyl cysteine. Minimal peptide length was set to six amino acids and a maximum of two miscleavages was allowed. The false discovery rate was set to 0.01 for peptide and protein identifications.

### Database

The protein expression levels of PD-L1 and the subunits of TRAPP complex were based on available proteomic analysis in human colorectal cancer tissues^16^. We analyzed specific protein expression in colorectal cancer using CPTAC Proteomics Data in the cBioPortal (http://www.cbioportal.org/)^30^.

### Statistical analysis

GraphPad Prism (7.03) software (GraphPad Inc., La Jolla, CA, USA) was used for the statistical analyses. Enumeration data were compared using a two-tailed Student’s *t* test or nonparametric Mann–Whitney test. The IHC scores were evaluated using Image-Pro Plus (6.0) and correlation assays were assessed using a Spearman correlation test. For IHC and immunofluorescence assays, we conducted at least three independent experimental replicates and the images were captured from five randomly selected fields under the microscope. The fluorescence intensity and colocalization of stained proteins were analyzed using ImageJ (2.1.0) software. The plot in Fig. 1g was produced using the Scatterstats tool in Hiplot (https://hiplot.com.cn). A *P* value <0.05 was considered statistically significant. All data were from at least two or three independent experiments and are shown as the means ± the standard deviation or means ± the standard error of the mean.

### Reporting summary

Further information on research design is available in the Nature Research Reporting Summary linked to this article.

## Supplementary information

Supplementary Information

Reporting summary

## Data Availability

The mass spectrometry proteomics data have been deposited to the ProteomeXchange Consortium via the PRIDE partner repository with the dataset identifier PXD027826. The proteomic analysis used in this study are available in CPTAC Proteomics Data in the cBioPortal (http://www.cbioportal.org/). The remaining data are available within the Article, Supplementary Information, or Source Data file. Source data are provided with this paper.

## Source data

Source data
