# Supplementary material for: TRAPPC4 regulates the intracellular trafficking of PD-L1 and antitumor immunity
Source: Nat Commun. 2021 Sep 13;12:5405. doi: 10.1038/s41467-021-25662-9 (PMC8438078; doi:10.1038/s41467-021-25662-9)
Supplement: Supplementary file 1 — Supplementary Information [file 41467_2021_25662_MOESM1_ESM.pdf]

TRAPPC4 regulates the intracellular trafficking of PD-L1 and anti-tumor immunity

Yimeng Ren[#1, 2, 3], Yun Qian[#1, 2, 3], Luoyan Ai[1,2, 3, 4], Yile Xie[1, 2, 3], Yaqi Gao[1, 2, 3], Ziyan Zhuang[1, 2, 3], Jinxian Chen[5], Ying-Xuan Chen[1, 2, 3], Jing-Yuan Fang[*1, 2, 3]

[1] State Key Laboratory for Oncogenes and Related Genes, Key Laboratory of Gastroenterology and Hepatology, Ministry of Health, 145 Middle Shandong Road, 200001 Shanghai, China [2] Division of Gastroenterology and Hepatology, Renji Hospital, School of Medicine, Shanghai Jiao Tong University, 145 Middle Shandong Road, 200001 Shanghai, China [3]Shanghai Institute of Digestive Disease, Renji Hospital, School of Medicine, Shanghai Jiao Tong University, 145 Middle Shandong Road, 200001 Shanghai, China [4] Department of Medical Oncology, Zhongshan Hospital, Fudan University, 180 FengLin Road, 200032, Shanghai, China [5] Division of Gastrointestinal Surgery, Renji Hospital, School of Medicine, Shanghai Jiao Tong University, 145 Middle Shandong Road, 200001 Shanghai, China
[#]Contributed equally.

Supplementary information

Table of Contents

Supplementary Figures 2

Supplementary Fig.1 Detection of PD-L1-binding TRAPP subunits by mass spectrometry. 2

Supplementary Fig. 2 Verification of the results of the mass spectrometry in RKO and HEK-293 cells. 3

Supplementary Fig. 3 Regulation of PD-L1 expression by subunits of the TRAPP complex. 4

Supplementary Fig. 4 The effects of TRAPPC4 and other TRAPP subunits on anti-tumor immunity. 6

Supplementary Fig. 5 Knockdown of TRAPPC4 affects tumor growth and tumor microenvironment components in vivo. 7

Supplementary Fig. 6 Regulation by TRAPPC4 and RAB11 on the expression of PD-L1. .. 8

Supplementary Fig. 7 Overexpression of TRAPPC4 promotes the efficacy of immune checkpoint blockade therapy. 9

Supplementary Fig. 8 Infiltration of the TILs in Trappc4-OE tumors with or without anti-PD-L1 treatment was analyzed with flow cytometry. 10

Supplementary Fig. 9 Scheme of the gating strategy used in flow cytometry assays. 11

Supplementary Tables 12

Supplementary Table 1. Proteomic analysis of co-expression of PD-L1 and the subunits of TRAPP complex (cohort 2) 12

Supplementary Table 2. Reagents or resources used in this study 12

Supplementary Table 3. Clinical and pathological information of 32 cases CRC patients (cohort 1) 15

Supplementary Table 4. Sequence of si-RNAs in this study 17

Supplementary Table 5. Primers for quantitative real-time PCR analysis 17

Supplementary Figures

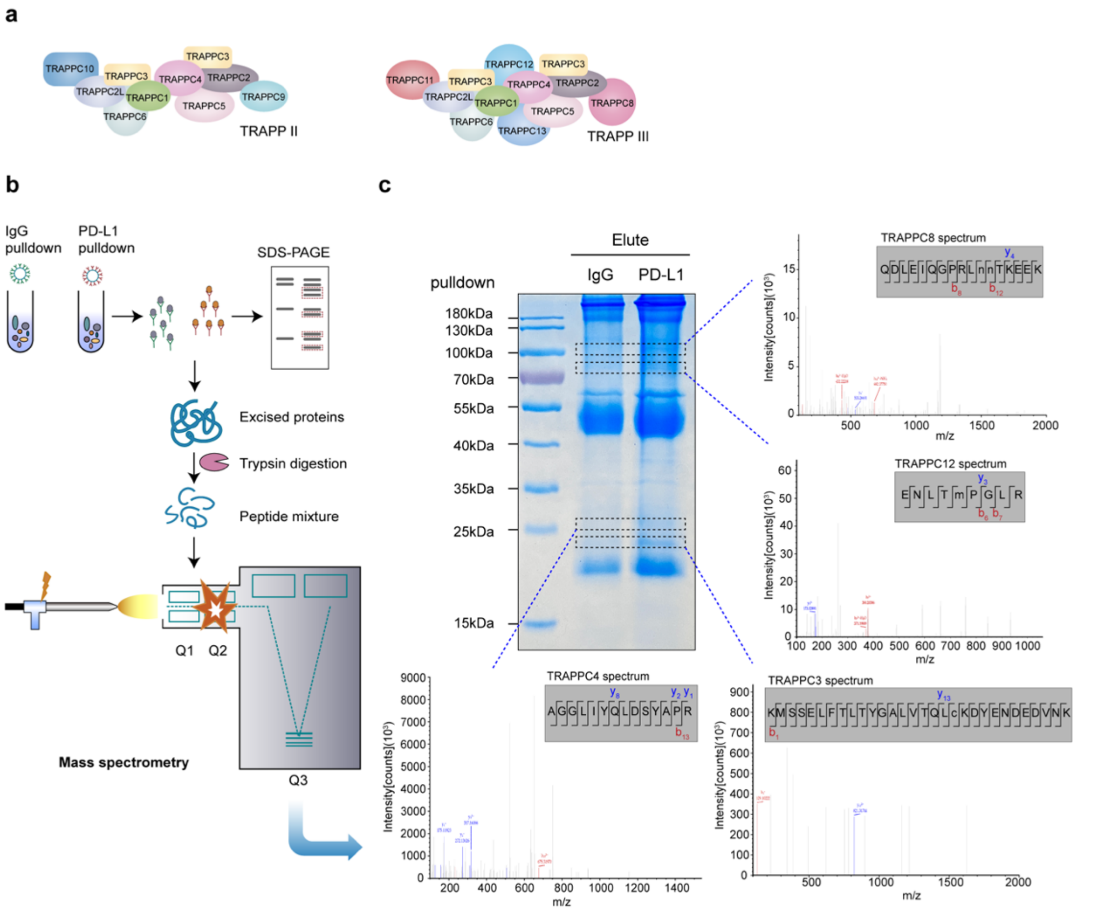

Supplementary Fig. 1 Detection of PD-L1-binding TRAPP subunits by mass spectrometry. a, Model of the mammalian TRAPP complexes. **b**, Schematic representation of the workflow to detect PD-L1-binding TRAPP subunits. The antibody targeting PD-L1 was added into a lysate of RKO cells to immunoprecipitated the endogenous PD-L1 protein along with its interacting proteins. Immunoprecipitation of IgG was included as the negative control. The proteins were separated by SDS-PAGE and stained by Coomassie brilliant blue to determine the specific stained bands in PD-L1 immunoprecipitation and analyzed using LC-MS/MS. **c**, Mass spectrometry analysis identified TRAPPC3, 4, 8, and 12 as the interaction partners of PD-L1. The bands indicated in this figure were analyzed using LC-MS/MS and the interacting TRAPP subunits were identified in presence of PD-L1 immunoprecipitation and absence of that of IgG. Source data are provided as a Source Data file.

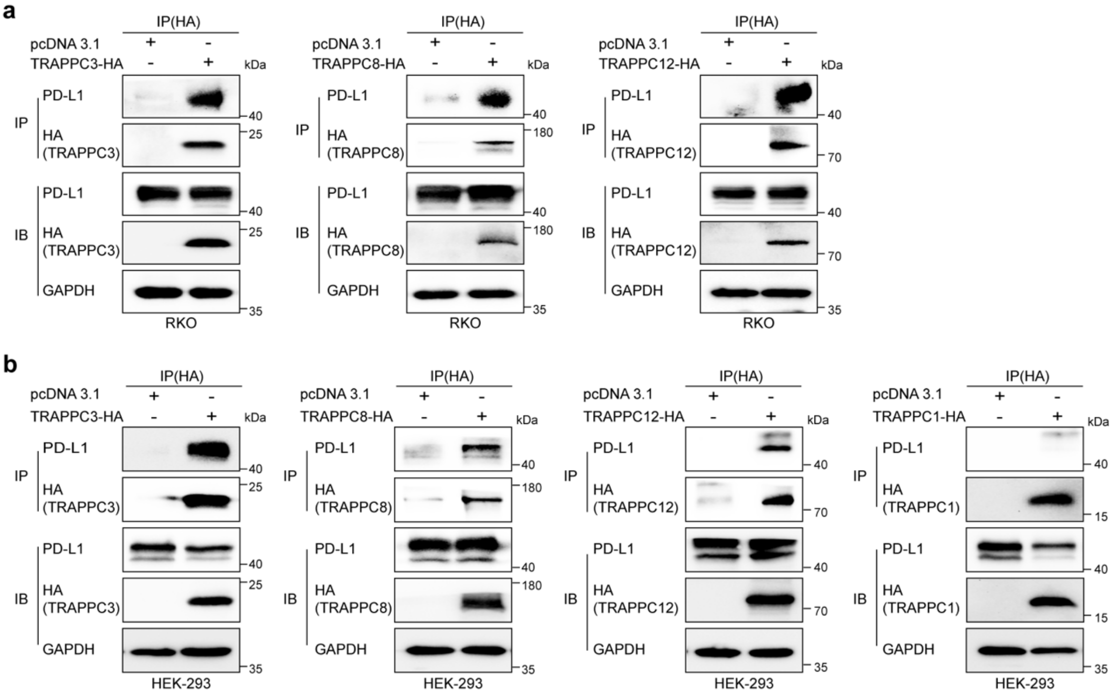

Supplementary Fig. 2 Verification of the results of the mass spectrometry in RKO and HEK-293 cells. a& b, The results of the mass spectrometry were verified by immunoprecipitation of the indicated TRAPP subunits in RKO (**a**) and HEK293 (**b**) cells. RKO and HEK293 cells were transfected with HA-TRAPPC3, 8, and 12 plasmids and immunoprecipitated with anti-HA antibodies to detect their interaction with PD-L1. HA-TRAPPC1 was included as a control representing the TRAPP subunits lacking an interaction with PD-L1. This experiment was repeated twice with similar results. Source data are provided as a Source Data file.

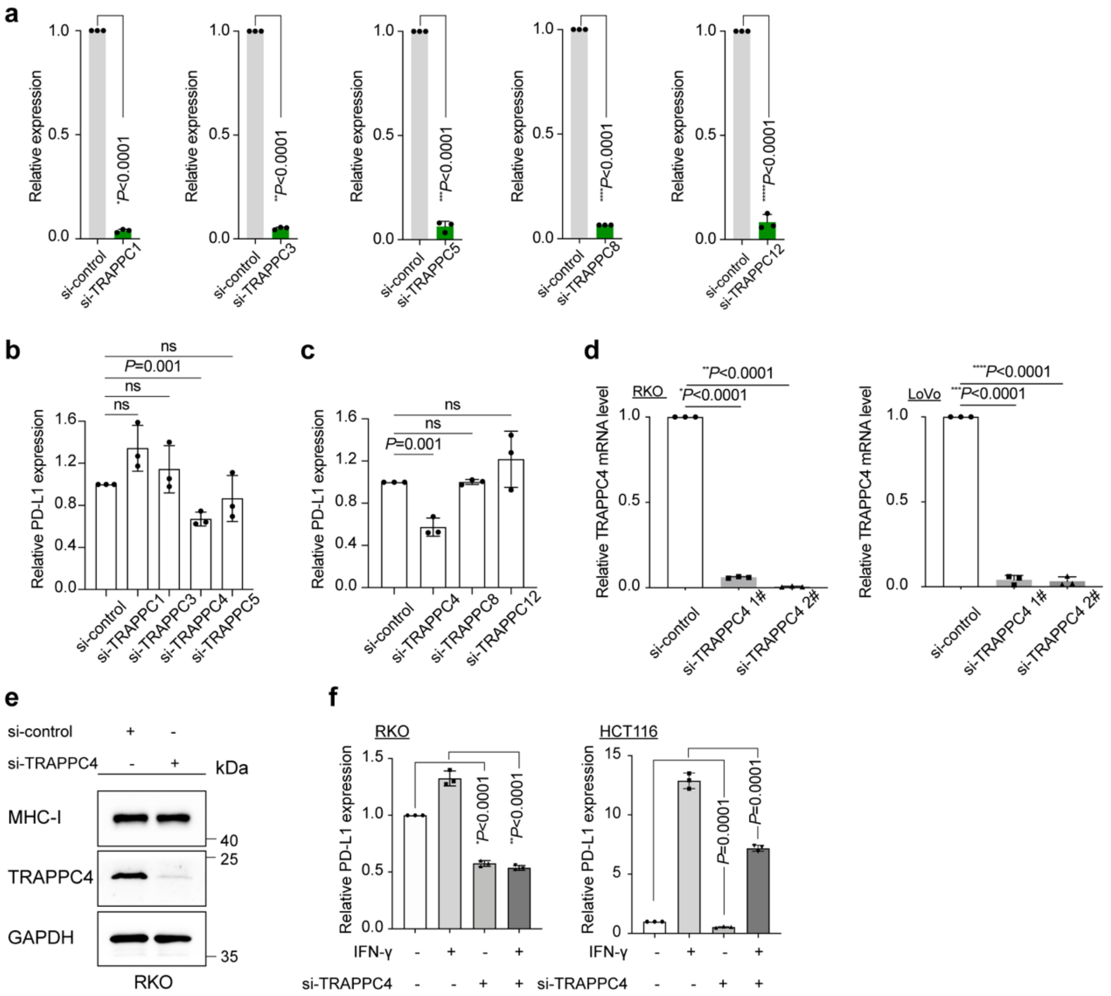

Supplementary Fig. 3 Regulation of PD-L1 expression by subunits of the TRAPP complex. a, The knockdown efficiency of siRNAs targeting different subunits of the TRAPP complex was determined using qRT-PCR in RKO cells. The means ± SD of three biological replicates is shown. ($^{*}P$=2.19e-9, $^{**}P$= 1.1e-9, $^{***}P$=3.69e-7, $^{****}P$=8e-15, $^{*****}P$=1.72e-6) **b& c**, The semiquantitative analysis of the indicated immunoblots in **Fig. 2b**. Values indicated means ± SD of the relative level of PD-L1 from three independent replicates, compared using a two-sided Student's *t* test. **d**, The knockdown efficiency of TRAPPC4 siRNA was determined using qRT-PCR in RKO cells and LoVo cells. The means ± SD of three biological replicates is shown. Data were analyzed by two-sided student's *t* test. ($^{*}P$=3.82e-10, $^{**}P$=6e-11, $^{***}P$=3.79e-7, $^{****}P$=4.13e-7) **e**, Western blot showing that interference with TRAPPC4 expression did not alter the level of MHC class I. The experiment was conducted three times independently with similar results. **f**, Values indicate means ± SD of the relative

gray values of the indicated blots from three independent assays, compared by a two-sided Student's *t* test. ($^{*}P$=7.93e-6, $^{**}P$=3.84e-5) Source data are provided as a Source Data file.

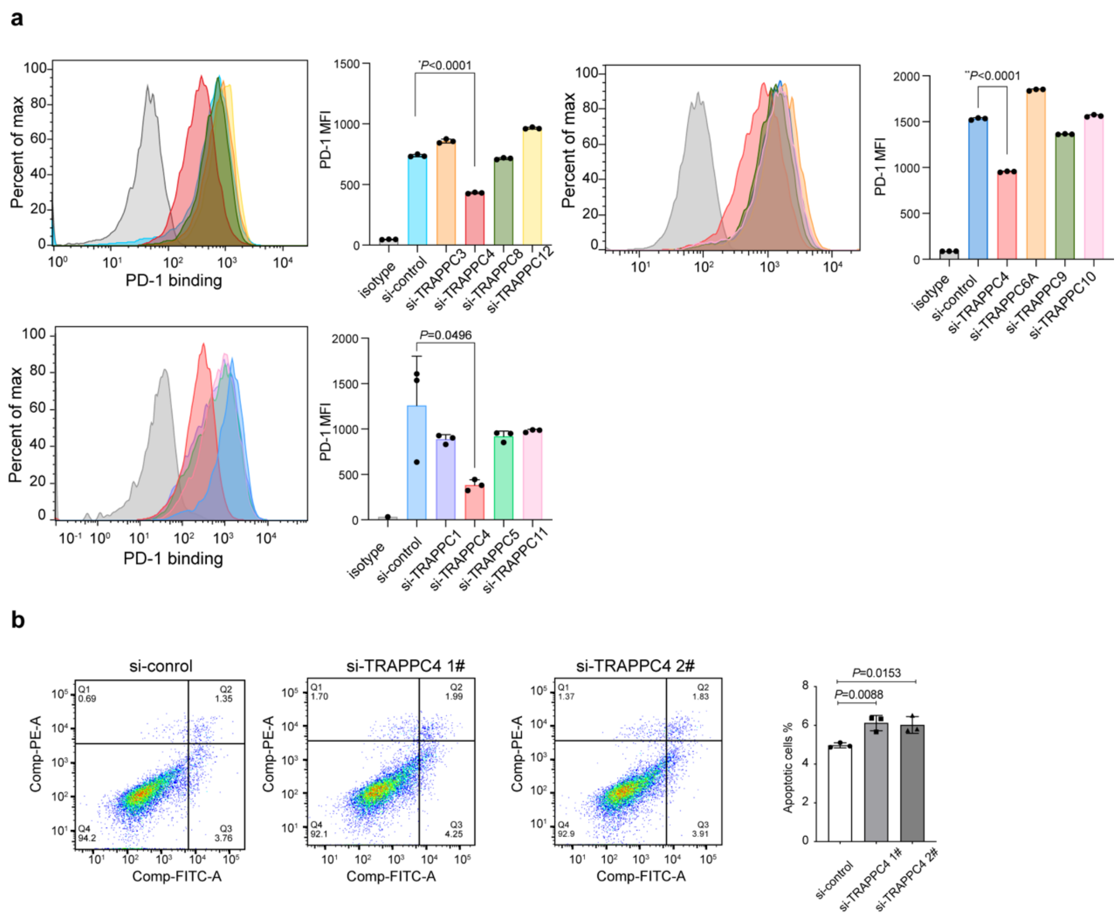

Supplementary Fig. 4 The effects of TRAPPC4 and other TRAPP subunits on anti-tumor immunity. a, The binding of PD-1 with PD-L1 on RKO cells treated with the indicated siRNAs was detected using flow cytometry. The y-axis indicates the MFI of fluorescence conjugated PD-1. This figure represents the result of three independent experimental replicates. Values indicate means ± SD, compared using a two-tailed Student's *t* test. ($^{*}P$=1.57e-6, $^{**}P$=1.77e-7) **b**, Flow cytometry assay indicating that a slight increase in apoptosis was induced by interference with TRAPPC4 expression. Values indicate means ± SD of the percentage of apoptotic cells from three independent replicates. Statistical analysis was performed using a two-tailed Student's *t* test. Source data are provided as a Source Data file.

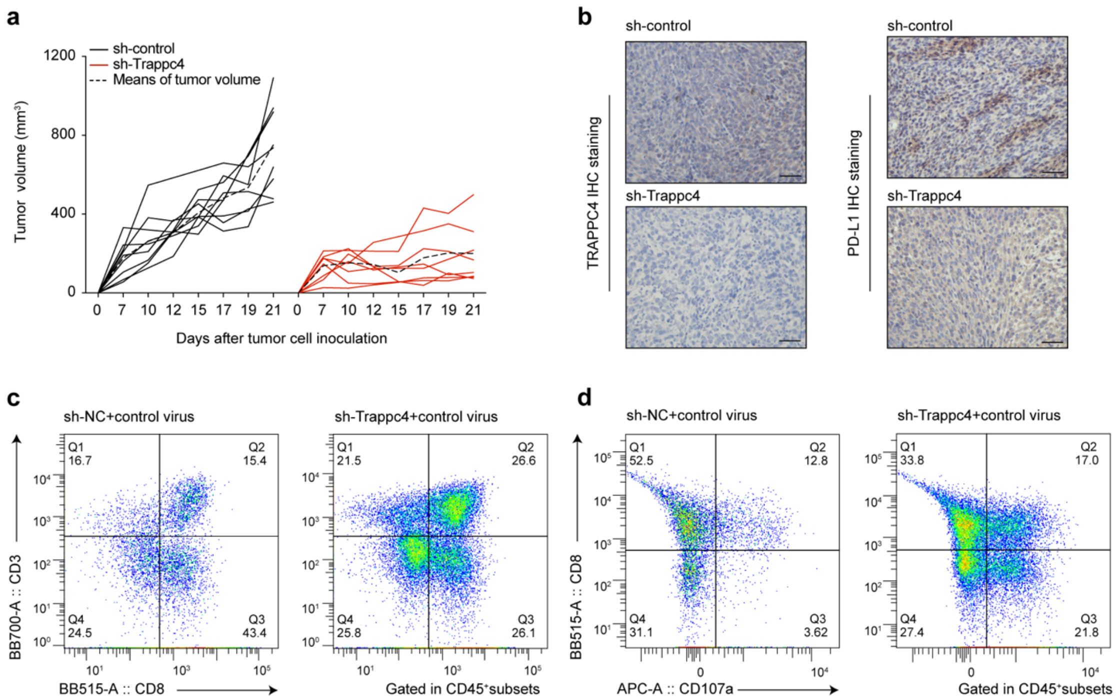

Supplementary Fig. 5 Knockdown of TRAPPC4 affects tumor growth and tumor microenvironment components in vivo. a, The growth of individual tumors in mice bearing control and Trappc4-deficient MC38 tumors (*n*=9 per group). The dashed lines represented the mean volumes of the tumors in the different groups. **b**, Representative immunohistochemical staining of TRAPPC4 and PD-L1 of tumors in different groups of mice. The experiment was repeated in three animal samples with similar results. Scale bars, 100μm. **c& d**, $CD3^+$ $CD8^+$ T cells (c) and $CD8^+$ $CD107a^+$ T cells (d) were analyzed using flow cytometry and the representative images with the percentage of the target subsets are shown, as gated in $CD45^+$ subsets. Source data are provided as a Source Data file.

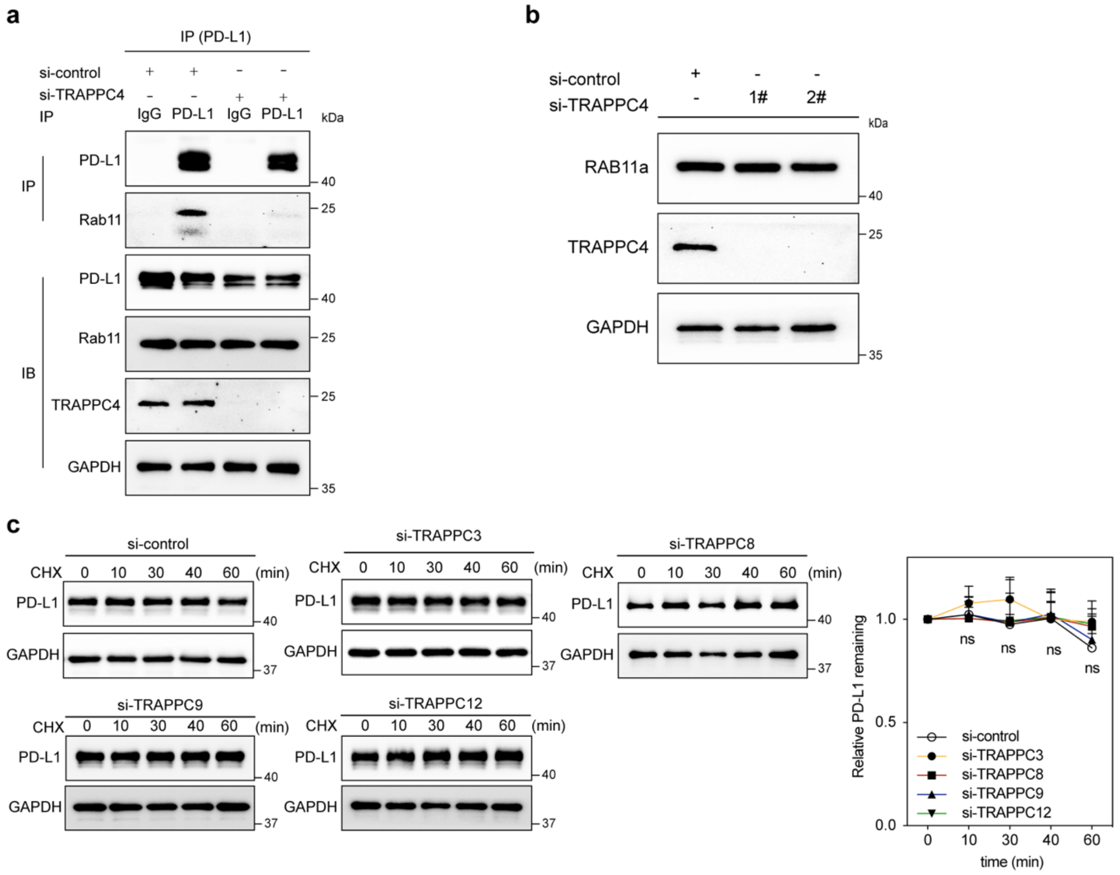

Supplementary Fig. 6 Regulation by TRAPPC4 and RAB11 on the expression of PD-L1. a, The Co-IP assay detecting interaction between PD-L1 and RAB11 with or without the knockdown of TRAPPC4. This experiment was repeated three times with similar results. **b**, Western blotting assay showing that knockdown of Trappc4 did not affect the expression of RAB11 in RKO cells. This experiment was repeated independently three times with similar results. **c**, CHX-chase assay indicating that the stability of PD-L1 was not significantly affected upon the knockdown of the indicated TRAPP subunits in RKO cells. The relative level of the remaining PD-L1 is quantified on the right and values indicates means ± SEM of the relative gray values. Statistical analysis was carried out using a two-tailed Student's *t*-test. This experiment was repeated three times with similar results. ns, not significant. Source data are provided as a Source Data file.

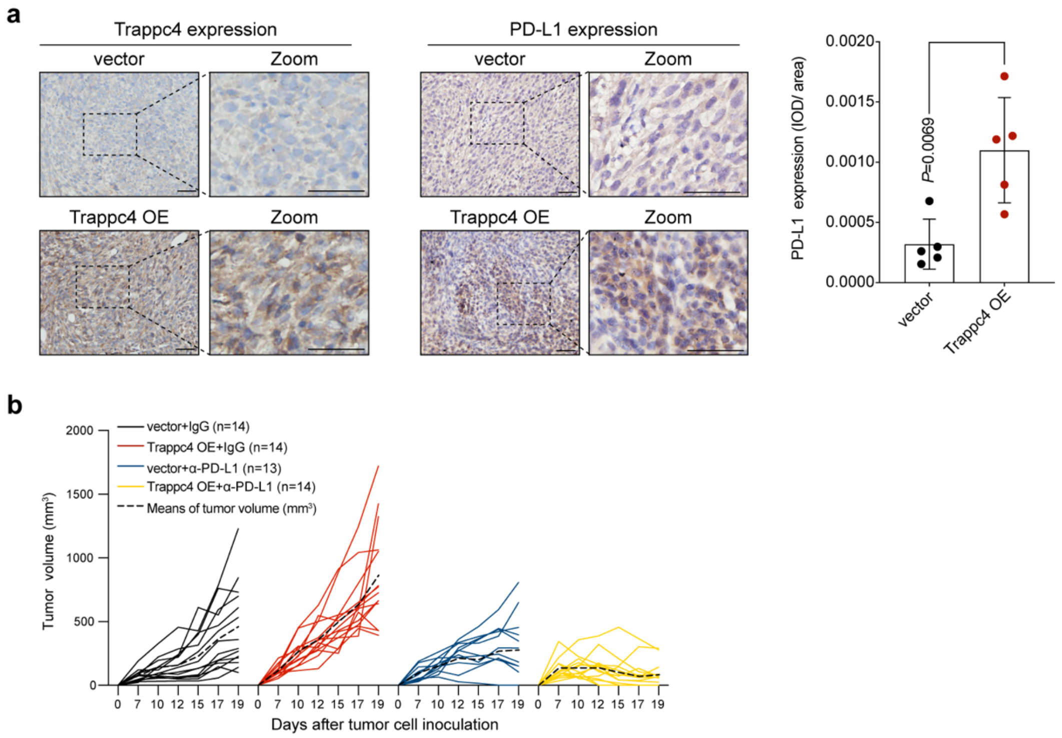

Supplementary Fig. 7 Overexpression of TRAPPC4 promotes the efficacy of immune checkpoint blockade therapy. a, Detection of TRAPPC4 and PD-L1 expression with immunohistochemical staining in control and Trappc4-OE groups. PD-L1 IHC scores were assessed using Image-Pro Plus and values indicates means ± SD from each group (n= 5 per group). Statistical analysis was carried out using a two-tailed Student's t-test. Scale bars, 100 µm. OE, overexpressing. **b**, The growth of individual tumors in the control and Trappc4-OE groups, with or without treatment with the anti-PD-L1 mAb. The dashed lines represent the mean volumes of the tumors in the different groups. Source data are provided as a Source Data file.

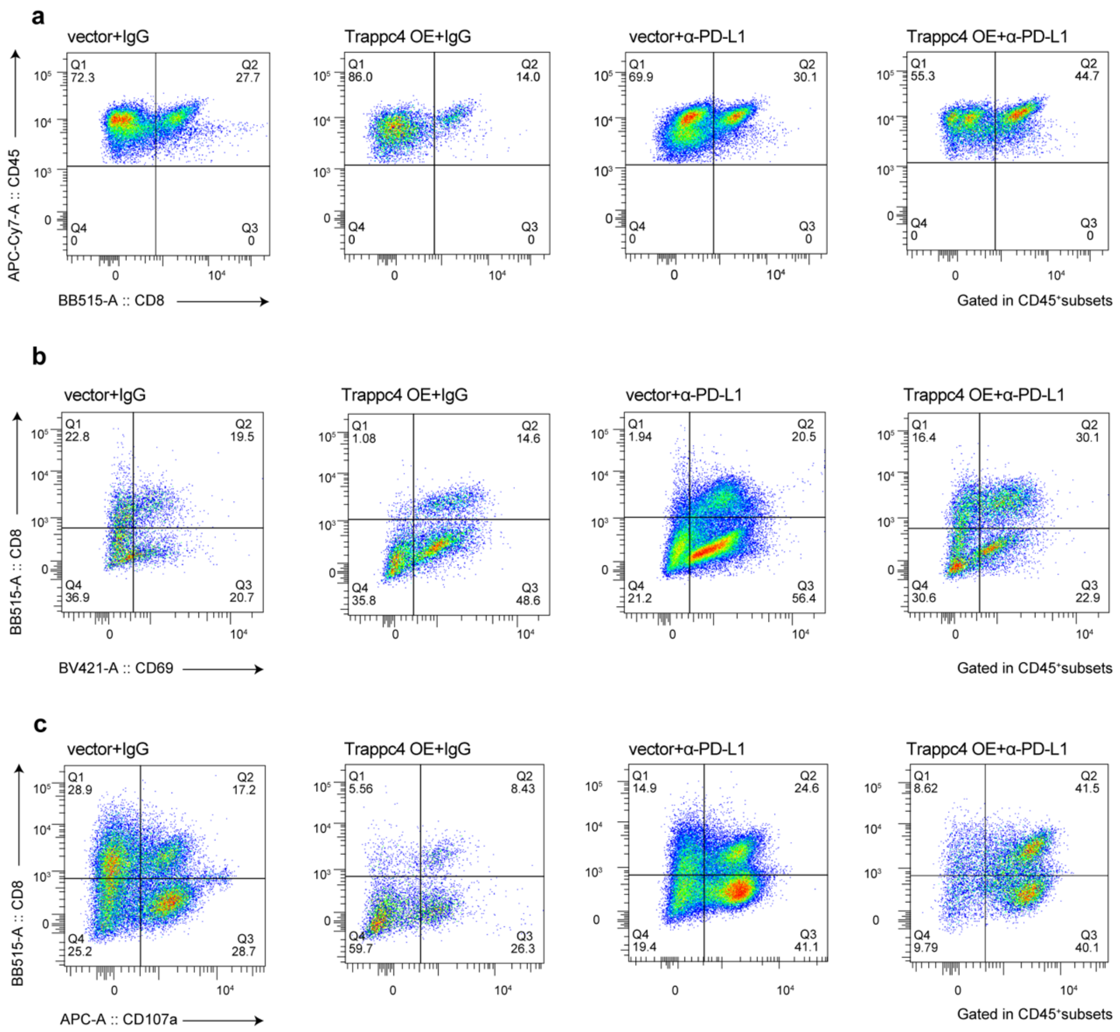

Supplementary Fig. 8 Infiltration of the TILs in Trappc4-OE tumors with or without anti-PD-L1 treatment was analyzed with flow cytometry. a-c, Infiltration of $CD8^+$ T cells (c), $CD8^+$ $C69^+$ T cells (d) $CD8^+$ $CD107a^+$ T cells (e) in different groups in **Fig. 6a** were analyzed using flow cytometry and the representative images with the percentage of the target subsets are shown, as gated in $CD45^+$ subsets. Source data are provided as a Source Data file.

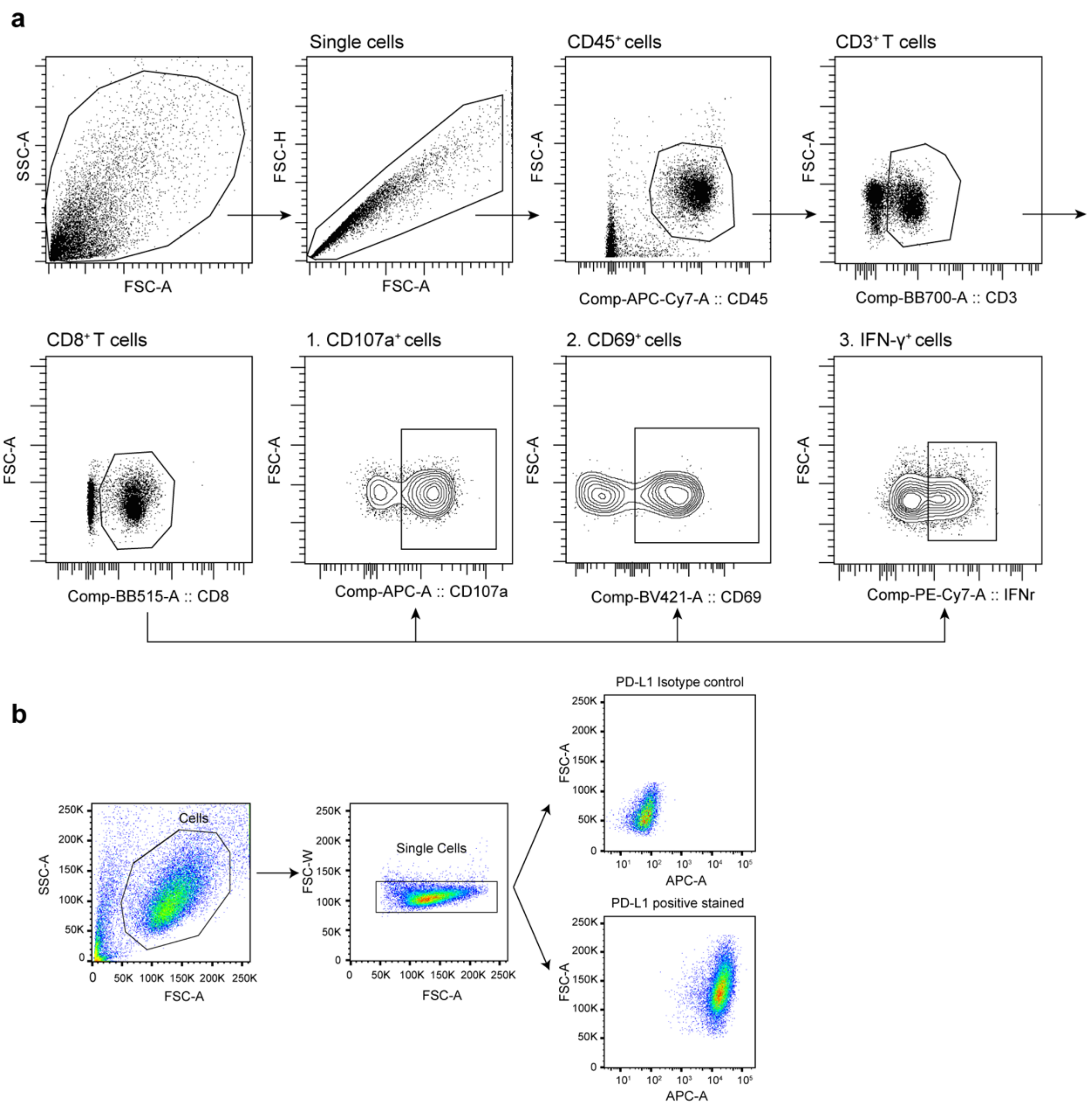

Supplementary Fig. 9 Scheme of the gating strategy used in flow cytometry assays. a, Scheme of the gating strategy used to examine different cell subsets in Fig. 3 and Fig. 6. **b**, Representative example of the gating strategies used for detection of PD-L1 on the cell surface.

Supplementary Tables

Supplementary Table 1. Proteomic analysis of co-expression of PD-L1 and the subunits of TRAPP complex (cohort 2)

Co-expression with PD-L1	Pearson r	*P*-value*
TRAPPC1	0.75	0.09
TRAPPC2	0.00	0.99
TRAPPC3	0.57	0.0045
TRAPPC4	0.69	0.0003
TRAPPC5	0.69	0.0003
TRAPPC6A	-0.05	0.91
TRAPPC6B	0.35	0.1
TRAPPC8	0.41	0.05
TRAPPC9	0.08	0.75
TRAPPC10	0.12	0.61
TRAPPC11	0.23	0.3
TRAPPC12	0.07	0.75
TRAPPC13	0.25	0.27

* Data were analyzed by two-sided Pearson correlation.

Supplementary Table 2. Reagents or resources used in this study

**Antibodies**			
**Name**	**Source**	**Catalogue no.**	**Dilution**
Rabbit anti- PD-L1	Cell signaling Technology	CAT#13684	1:1,000 (WB)<br>1:100 (IHC)
Rabbit anti-PD-L1	Proteintech	CAT#17952-1-AP	1:100 (IF, IHC)
Mouse anti-PD-L1	Cell signaling Technology	CAT#29122	1:100 (IF)
Mouse anti-TRAPPC4	Abcam	CAT#ab57364	1:1,000 (WB)
Mouse anti-TRAPPC4	Abnova	CAT#H00051399-M01	1:1,000 (WB)<br>1:100 (IF)
Rabbit anti-TRAPPC4	Sigma-Aldrich	CAT#HPA041371	1:1,000 (WB)<br>1:100 (IHC)
Rabbit anti-CD8	Cell signaling Technology	CAT#98941	1:200
Rabbit anti-CD4	Abcam	CAT#ab183685	1:200
Rabbit anti-Giantin	Abcam	CAT#ab80864	1:200
Rabbit anti-EEA1	Abcam	CAT#ab2900	1:200
Mouse anti-Lamp1	Abcam	CAT#ab25630	1:200
Mouse anti-Rab11	BD Biosciences	CAT#610656	1:1,000

Mouse anti-HA tag	Biolegend	CAT#901513	1:1,000
Anti-GAPDH-HRP	KANGCHENG Technology	CAT#KC-5G5	1:3,000
Anti-rabbit-HRP	KANGCHENG Technology	CAT#KC-RB-035	1:5,000
Anti-mouse-HRP	KANGCHENG Technology	CAT#KC-MM-035	1:5,000
Anti-mouse Alexa Fluor 488 dye conjugated antibody	Invitrogen	CAT#A-21202	1:400
Anti-rabbit Alexa Fluor 594 dye conjugated antibody	Invitrogen	CAT#R37119	1:400
Anti-human Alexa Fluor 488 dye conjugated antibody	Invitrogen	CAT#A-11013	1:400
APC anti-human CD274 (B7-H1, PD-L1) Antibody	Biolegend	CAT#329708	1:250
APC mouse anti-human CD45	BD Biosciences	CAT#561863	1:200
APC-Cy7 Rat anti-mouse CD45	BD Biosciences	CAT#557659	1:100
PerCP Cy5.5 anti-mouse CD3	eBioscience	CAT#45-0031-82	1:100
FITC anti-mouse CD8a	Biolegend	CAT#100706	1:100
BV421 anti-mouse CD69	Biolegend	CAT#104528	1:100
APC anti-mouse CD107a	Biolegend	CAT#121614	1:100
PE-Cy7 Rat anti-mouse IFN-γ	BD Biosciences	CAT#557649	1:50

**Chemicals, reagents and Recombinant Proteins**		
**Name**	**Source**	**Catalogue no.**
AOM	Sigma-Aldrich	CAT# A5486
DSS	MP Chemicals Inc	CAT# 0216011080
FuGENE HD	Promega	CAT#E2311
DharmaFECT Transfection Reagent	Dharmacon	CAT#T-2001-02
DharmaFECT Duo Transfection Reagent	Dharmacon	CAT#T-2010-03

Recombinant human PD-1 Fc chimera protein	R&D Systems	CAT#1086-PD-050
Recombinant Human IFN-gamma Protein	R&D Systems	CAT#285-IF-100/CF
Cycloheximide	Sigma-Aldrich	CAT#C1988
Chloroquine	Sigma-Aldrich	CAT#C6628
NH_4Cl	Aladdin Chemistry	CAT#A116373
MG132	Sigma-Aldrich	CAT# M8699
RNAiso Plus	Takara	CAT# 9109
DAPI	SouthernBiotech	CAT#0100-20
Pierce IP Lysis Buffer	Thermo Fisher	CAT#87787
Pierce protein G Agarose	Thermo Fisher	CAT#20398
InVivoMab anti-mouse PD-L1	Bioxcell	CAT#BE0101
**Critical Commercial Assays**		
**Name**	**Source**	**Catalogue no.**
FITC Annexin V Apoptosis Detection Kit I	BD Biosciences	CAT#556547
PrimeScript RT Reagent Kit	Takara	CAT# RR036A
TB Green Premix Ex Taq II	Takara	CAT# RR820L
Pierce BCA protein assay kit	Thermo Fisher	CAT#23227
T Cell Activation/Expansion Kit	Miltenyi Biotec	CAT#130-091-441
**Recombinant DNA**		
**Name**	**Source**	**Catalogue no.**
pcDNA-Myc-TRAPPC4	GeneRay, Inc	N/A
pcDNA-Flag-PD-L1	GeneRay, Inc	N/A
pcDNA-HA-Rab11	GeneRay, Inc	N/A
pcDNA-HA-Rab11Q70L	GeneRay, Inc	N/A
pcDNA-HA-Rab11S25N	GeneRay, Inc	N/A
**Experimental Models: Cell Lines**		
**Name**	**Source**	**Catalogue no.**
HCT116 Cells	ATCC	CAT# CCL-247
RKO Cells	ATCC	CAT# CRL-2577
LoVo Cells	ATCC	CAT# CCL-229
MC38	BMCR	CAT# 1101MOU-PUMC000523
**Experimental Models: Strains**		
TRAPPC4$^{\Delta IEC}$		N/A
Escherichia coli strain *DH5α*	TIANGEN	CAT#CB101
**Biological Samples**		

Formalin-fixed paraffin-embedded colorectal cancer tissues	Renji Hospital affiliated with Shanghai Jiaotong University School of Medicine	N/A
**Software and Algorithms**		
ImageJ 2.1.0	National Institutes of Health (open source)	https://imagej.nih.gov/ij/
Image-Pro Plus 6.0	Media Cybernetics, Inc.	https://www.mediacy.com/imagepro plus
Graphpad Prism 7.03	Graphpad	https://www.graphpad.com/ scientific-software/prism/
ZEN 2011	ZEISS	https://www.zeiss.com/microscopy/
FlowJo 10.4	BD Biosciences	https://www.flowjo.com
FACSDiva 6.2	BD Biosciences	https://www.bdbiosciences.com/en-us/products/software/instrument-software/bd-facsdiva-software

Supplementary Table 3. Clinical and pathological information of 32 cases CRC patients (cohort 1)

NO.	Location	Tumor Size (cm)	Grade	TNM stage	Expression of TRAPPC4 (IOD/area)	Expression of PD-L1 (IOD/area)
1	Descending colon	4*3*0.6	II	T4N1M1	9.57	9.21
2	Sigmoid colon	2.5*1.5*0.8	II	T4N0M0	8.97	8.27
3	Sigmoid colon	4*4*1	II	T3N0M0	4.18	7.30
4	Rectum	4*3.5*1	II	T4aN0M0	11.40	12.45
5	Rectum	5*4*0.8	II	T3N0M0	7.02	4.29
6	Ileocecal junction	5.5*2*1.5	III	T4aN0M0	7.55	3.44
7	Sigmoid colon	4*3.5*0.8	II	T3N1M0	6.24	8.86
8	Ascending colon	4*3*1	II	T3N2M0	3.65	2.94

9	Sigmoid colon	4*3*1	II	T3N0M0	2.97	9.44
10	Ascending colon	5*4*4	II	T3N0M0	6.07	6.58
11	Ileocecal junction	4*4*3	II	T3N0M0	2.64	4.99
12	Rectum	3.5*2*0.5	II	T4aN0M0	3.84	6.98
13	Descending colon	5*5*2.5	II	T4aN0M0	0.03	0.06
14	Transverse colon	6*4*1	II	T3N0M0	12.50	7.29
15	Ileocecal junction	7*7*3	II-III	T3N0M0	14.40	8.78
16	Rectum	3*3*1	II	T4N0M0	12.11	9.15
17	Rectum	5*3.5*1	II	T4N2M0	17.20	24.30
18	Sigmoid colon	7*4*2	II	T4N0M0	9.84	13.31
19	Sigmoid colon	2.5*2*1.2	II	T4N2M1	2.56	0.01
20	Descending colon	6.5*4*3.5	II	T3N0M1	0.00	0.09
21	Ileocecal junction	5*3.5*0.7	II	T3N1M0	0.00	0.11
22	Rectum	5*3*1	II	T4N0M0	3.46	2.74
23	Sigmoid colon	7.5*3.5*3	II	T4N2M1	3.65	3.37
24	Descending colon	3.5*2.5*1	II-III	T4bN1bM0	0.00	0.43
25	Ascending colon	3.5*3*2	II	T3N1aM0	1.43	0.14
26	Rectum	7.5*5*1	II	T3N0M0	0.07	0.06
27	Ascending colon	8*6*1	II	T3N2M1	11.70	15.41
28	Rectum	3.5*1.5*1	II	T4N2M0	10.10	5.04
29	Rectum	7*5.5*2	II	T4N1M0	1.21	1.05
30	Ascending colon	3.5*3*1.5	II	T2N0M0	3.72	2.61
31	Rectum	2.5*2*0.7	II	T2N1M0	0.04	0.23
32	Rectum	4*3*0.5	II	T4N0M0	4.84	4.12

Supplementary Table 4. Sequence of si-RNAs in this study

Target name	Sense (5'-3')	Antisense (5'-3')
TRAPPC4-1	CGCCUCACUUCUAAUGAGATT	UCUCAUUAGAAGUGAGGCGTT
TRAPPC4-2	GGAUCAAGUUUGUGGUUCUTT	AGAACCACAAACUUGAUCCTT
TRAPPC1	GCUCCCGACUGGACUCCUATT	UAGGAGUCCAGUCGGGAGCTT
TRAPPC3	GAGACGGUGUGACAGAAAUTT	AUUUCUGUCACACCGUCUCTT
TRAPPC5	CGCUCAUCAACACCUACAUTT	AUGUAGGUGUUGAUGAGCGTT
TRAPPC6	CGGAUACUGUGUUGUUUGATT	UCAAACAACACAGUAUCCGTT
TRAPPC8	GGGUUGCUAUUUACUUAAATT	UUUAAGUAAAUAGCAACCCTT
TRAPPC9	GGCAUGUGAAACUAUUGAATT	UUCAAUAGUUUCACAUGCCTT
TRAPPC10	CUCCGAAGAUGAUUCACCUTT	AGGUGAAUCAUCUUCGGAGTT
TRAPPC11	GGGCCUGGAUGUAGUUUAUTT	AUAAACUACAUCCAGGCCCTT
TRAPPC12	GAUGCAGUUAAAGACUUGATT	UCAAGUCUUUAACUGCAUCTT
RAB11A-1	GGAGAUUCUGGUGUUGGAATT	UUCCAACACCAGAAUCUCCTT
RAB11A-2	GGAGCUGUAGGUGCCUUAUTT	AUAAGGCACCUACAGCUCCTT
RAB11A-3	CCUAGACUCUACAAAUGUATT	UACAUUUGUAGAGUCUAGGTT

Supplementary Table 5. Primers for quantitative real-time PCR analysis

Gene name	Forward	Reverse
TRAPPC1	TTCCAAACTAGCCGTTACAAACT	GCTCCACATACAGCGCACT
TRAPPC3	ACATTGGAGTCCGGCTGATTG	CCGCAGTTTCCCGAAAGTCAT
TRAPPC4	GTTCCACTCGCTCTTTGCCA	CCAGCTTGCCTAGGATCTGC
TRAPPC5	GTGGGTTTCTCCCCAGTGTAG	TTGAGCGTGCTGTTCTCCTT
TRAPPC8	GAAACCTTTCTTCAGTCGATGCC	ACGCTACCAACATACATGCTAAA
TRAPPC12	CACAGGAGTCGCTGGATAGAC	CTGCTAAGCCTTGCTCCAAAT
PD-L1	TGGCATTTGCTGAACGCATTT	TGCAGCCAGGTCTAATTGTTTT
GAPDH	GCATTGCCCTCAACGACCAC	CCACCACCCTGTTGCTGTAG
