# Supplementary material for: TRAPPC4 regulates the intracellular trafficking of PD-L1 and antitumor immunity
Source: Nat Commun. 2021 Sep 13;12:5405. doi: 10.1038/s41467-021-25662-9 (PMC8438078; doi:10.1038/s41467-021-25662-9)
Supplement: Supplementary file 2 — Reporting summary [file 41467_2021_25662_MOESM2_ESM.pdf]

nature portfolio

Corresponding author(s): Jing-Yuan Fang

Last updated by author(s): Aug 20, 2021

Reporting Summary

Nature Portfolio wishes to improve the reproducibility of the work that we publish. This form provides structure for consistency and transparency in reporting. For further information on Nature Portfolio policies, see our Editorial Policies and the Editorial Policy Checklist.

Statistics

For all statistical analyses, confirm that the following items are present in the figure legend, table legend, main text, or Methods section.

n/a	Confirmed	
☐	☒	The exact sample size (n) for each experimental group/condition, given as a discrete number and unit of measurement
☐	☒	A statement on whether measurements were taken from distinct samples or whether the same sample was measured repeatedly
☐	☒	The statistical test(s) used AND whether they are one- or two-sided<br>*Only common tests should be described solely by name; describe more complex techniques in the Methods section.*
☐	☒	A description of all covariates tested
☐	☒	A description of any assumptions or corrections, such as tests of normality and adjustment for multiple comparisons
☐	☒	A full description of the statistical parameters including central tendency (e.g. means) or other basic estimates (e.g. regression coefficient) AND variation (e.g. standard deviation) or associated estimates of uncertainty (e.g. confidence intervals)
☐	☒	For null hypothesis testing, the test statistic (e.g. F, t, r) with confidence intervals, effect sizes, degrees of freedom and P value noted<br>*Give P values as exact values whenever suitable.*
☒	☐	For Bayesian analysis, information on the choice of priors and Markov chain Monte Carlo settings
☒	☐	For hierarchical and complex designs, identification of the appropriate level for tests and full reporting of outcomes
☐	☒	Estimates of effect sizes (e.g. Cohen's d, Pearson's r), indicating how they were calculated

Our web collection on statistics for biologists contains articles on many of the points above.

Software and code

Policy information about availability of computer code

Data collection	Data of flow cytometry assays were collected by FACSDiva (version 6.2) software. Proteomic data of specific protein in colorectal cancer were analyzed using CPTAC Proteomics Data in the cBioPortal.
Data analysis	We performed the statistical analysis using version 7.03 of GraphPad Prism (GraphPad Software) for the unpaired, two-tailed Student's t test, nonparametric Mann–Whitney test and calculation of Pearson's correlation coefficient (R). Scores of immunohistochemical staining were evaluated with version 6.0 of Image-Pro Plus. The gray values of the specific immunoblots were measured with Image J software (2.1.0). The immunofluorescence micrographs were captured with ZEN system 2011 Black Edition and colocalization analysis was performed using "colocalization" plugin of Image J software. Data of flow cytometry assays were collected by FACSDiva (version 6.2) software and analyzed by FlowJo 10.4 (BD bioscience).

For manuscripts utilizing custom algorithms or software that are central to the research but not yet described in published literature, software must be made available to editors and reviewers. We strongly encourage code deposition in a community repository (e.g. GitHub). See the Nature Portfolio guidelines for submitting code & software for further information.

Data

Policy information about availability of data

All manuscripts must include a data availability statement. This statement should provide the following information, where applicable:

- Accession codes, unique identifiers, or web links for publicly available datasets
- A description of any restrictions on data availability
- For clinical datasets or third party data, please ensure that the statement adheres to our policy

The mass spectrometry proteomics data have been deposited to the ProteomeXchange Consortium via the PRIDE partner repository with the dataset identifier

PXD027826. The proteomic analysis used in this study are avsilable in CPTAC Proteomics Data in the cBioPortal (http://www.cbioportal.org/). The remaining data are available within the Article, Supplementary Information or Source Data file.

Field-specific reporting

Please select the one below that is the best fit for your research. If you are not sure, read the appropriate sections before making your selection.

- [x] Life sciences
- [ ] Behavioural & social sciences
- [ ] Ecological, evolutionary & environmental sciences

For a reference copy of the document with all sections, see nature.com/documents/nr-reporting-summary-flat.pdf

Life sciences study design

All studies must disclose on these points even when the disclosure is negative.

Sample size	Sample size estimates has been performed to obtain statistical significance and reproducibility. For in vitro experiments such as Western blot, qPCR, IHC and flow cytometry, at least three samples were used per group for minimal statistics requirements. For in vivo studies, the number of samples assigned to each treatment was selected to provide sufficient statistical power to discern significant differences in different groups.
Data exclusions	The only data points excluded were some of the tumor volume data that were clear outliers due to measuring errors in subcutaneous tumor models. For representation of the tumor growth, we included at least five tumor volumes per group in each measurement.
Replication	All experiments were performed multiple times independently as indicated in the figure legends, and all attempts at replication were successful.
Randomization	For in vivo studies using TrappcƵ IEC mice and their littermates, Trappc4flox mice, no randomization was performed due to their different genetic backgrounds. For subcutaneous tumor models, mice were randomized to different groups receiving the indicated MC38 cells. Mice inoculated with Trappc4OE MC38 cells or the control cells were randomized based on tumor volumes to treatment with anti-PD-L1 mAb or IgG respectively. Mice were age and sex matched.
Blinding	The capture process and evaluation of the H&E and immunohistochemical staining were performed by 2 individuals who were blinded as to treatments of different groups. In vivo studies were not performed in a blinded manner, since the investigators needed to know the treatment of each group to complete the studies. All experiments were assigned into groups with relevant controls. Data were collected objectively, and analyzed using software with objective standards.

Behavioural & social sciences study design

All studies must disclose on these points even when the disclosure is negative.

Study description	*Briefly describe the study type including whether data are quantitative, qualitative, or mixed-methods (e.g. qualitative cross-sectional, quantitative experimental, mixed-methods case study).*
Research sample	*State the research sample (e.g. Harvard university undergraduates, villagers in rural India) and provide relevant demographic information (e.g. age, sex) and indicate whether the sample is representative. Provide a rationale for the study sample chosen. For studies involving existing datasets, please describe the dataset and source.*
Sampling strategy	*Describe the sampling procedure (e.g. random, snowball, stratified, convenience). Describe the statistical methods that were used to predetermine sample size OR if no sample-size calculation was performed, describe how sample sizes were chosen and provide a rationale for why these sample sizes are sufficient. For qualitative data, please indicate whether data saturation was considered, and what criteria were used to decide that no further sampling was needed.*
Data collection	*Provide details about the data collection procedure, including the instruments or devices used to record the data (e.g. pen and paper, computer, eye tracker, video or audio equipment) whether anyone was present besides the participant(s) and the researcher, and whether the researcher was blind to experimental condition and/or the study hypothesis during data collection.*
Timing	*Indicate the start and stop dates of data collection. If there is a gap between collection periods, state the dates for each sample cohort.*
Data exclusions	*If no data were excluded from the analyses, state so OR if data were excluded, provide the exact number of exclusions and the rationale behind them, indicating whether exclusion criteria were pre-established.*
Non-participation	*State how many participants dropped out/declined participation and the reason(s) given OR provide response rate OR state that no participants dropped out/declined participation.*
Randomization	*If participants were not allocated into experimental groups, state so OR describe how participants were allocated to groups, and if allocation was not random, describe how covariates were controlled.*

Ecological, evolutionary & environmental sciences study design

All studies must disclose on these points even when the disclosure is negative.

Study description	*Briefly describe the study. For quantitative data include treatment factors and interactions, design structure (e.g. factorial, nested, hierarchical), nature and number of experimental units and replicates.*
Research sample	*Describe the research sample (e.g. a group of tagged Passer domesticus, all Stenocereus thurberi within Organ Pipe Cactus National Monument), and provide a rationale for the sample choice. When relevant, describe the organism taxa, source, sex, age range and any manipulations. State what population the sample is meant to represent when applicable. For studies involving existing datasets, describe the data and its source.*
Sampling strategy	*Note the sampling procedure. Describe the statistical methods that were used to predetermine sample size OR if no sample-size calculation was performed, describe how sample sizes were chosen and provide a rationale for why these sample sizes are sufficient.*
Data collection	*Describe the data collection procedure, including who recorded the data and how.*
Timing and spatial scale	*Indicate the start and stop dates of data collection, noting the frequency and periodicity of sampling and providing a rationale for these choices. If there is a gap between collection periods, state the dates for each sample cohort. Specify the spatial scale from which the data are taken*
Data exclusions	*If no data were excluded from the analyses, state so OR if data were excluded, describe the exclusions and the rationale behind them, indicating whether exclusion criteria were pre-established.*
Reproducibility	*Describe the measures taken to verify the reproducibility of experimental findings. For each experiment, note whether any attempts to repeat the experiment failed OR state that all attempts to repeat the experiment were successful.*
Randomization	*Describe how samples/organisms/participants were allocated into groups. If allocation was not random, describe how covariates were controlled. If this is not relevant to your study, explain why.*
Blinding	*Describe the extent of blinding used during data acquisition and analysis. If blinding was not possible, describe why OR explain why blinding was not relevant to your study.*

Did the study involve field work? ☐ Yes ☐ No

Field work, collection and transport

Field conditions	*Describe the study conditions for field work, providing relevant parameters (e.g. temperature, rainfall).*
Location	*State the location of the sampling or experiment, providing relevant parameters (e.g. latitude and longitude, elevation, water depth).*
Access & import/export	*Describe the efforts you have made to access habitats and to collect and import/export your samples in a responsible manner and in compliance with local, national and international laws, noting any permits that were obtained (give the name of the issuing authority, the date of issue, and any identifying information).*
Disturbance	*Describe any disturbance caused by the study and how it was minimized.*

Reporting for specific materials, systems and methods

We require information from authors about some types of materials, experimental systems and methods used in many studies. Here, indicate whether each material, system or method listed is relevant to your study. If you are not sure if a list item applies to your research, read the appropriate section before selecting a response.

Materials & experimental systems

n/a	Involved in the study	
☐	☒	Antibodies
☐	☒	Eukaryotic cell lines
☒	☐	Palaeontology and archaeology
☐	☒	Animals and other organisms
☐	☒	Human research participants
☒	☐	Clinical data
☒	☐	Dual use research of concern

Methods

n/a	Involved in the study	
☒	☐	ChIP-seq
☐	☒	Flow cytometry
☒	☐	MRI-based neuroimaging

Antibodies

Antibodies used	Western blot primary antibodies:<br>Rabbit anti- PD-L1 (CAT#13684, CST), 1:1,000 dilution<br>Mouse anti-Trappc4 (CAT#ab57364, abcam; CAT#H00051399-M01, clone 2D10, Abnova), 1:1,000 dilution<br>Rabbit anti-Trappc4 (CAT#HPA041371, Sigma-Aldrich), 1:1,000 dilution<br>Mouse anti-Rab11 (CAT#610656, BD Biosciences), 1:1,000 dilution<br>Mouse anti-HA tag (CAT#901513, Biolegend), 1:1,000 dilution<br>Anti-GAPDH (CAT#KC-5G5, KANGCHENG Technology), 1: 3,000 dilution<br><br>Western blot secondary antibodies:<br>Anti-rabbit-HRP (CAT#KC-RB-035, KANGCHENG Technology), 1:5,000 dilution<br>Anti-mouse-HRP (CAT#KC-MM-035, KANGCHENG Technology), 1:5,000 dilution<br><br>Immunofluorescence primary antibodies:<br>Rabbit anti-PD-L1 (CAT#17952-1-AP, Proteintech), 1:100 dilution<br>Mouse anti-PD-L1 (CAT#29122, CST), 1:100 dilution<br>Mouse anti-Trappc4 (CAT#H00051399-M01, Abnova), 1:100 dilution<br>Rabbit anti-Giantin (CAT#ab80864, abcam), 1:200 dilution<br>Rabbit anti-EEA1 (CAT#ab2900, abcam), 1:200 dilution<br>Mouse anti-Lamp1 (CAT#ab25630, abcam), 1:200 dilution<br><br>Immunofluorescence secondary antibodies:<br>Anti-mouse Alexa Fluor 488 dye conjugated antibody (CAT#A-21202, Invitrogen), 1:400 dilution<br>Anti-rabbit Alexa Fluor 594 dye conjugated antibody (CAT#R37119, Invitrogen), 1:400 dilution<br><br>IHC antibodies:<br>Rabbit anti- PD-L1 (CAT#13684, CST), 1:100 dilution<br>Rabbit anti-Trappc4 (CAT#HPA041371, Sigma-Aldrich), 1:100 dilution<br>Rabbit anti-CD8 (CAT#98941, CST), 1:200 dilution<br>Rabbit anti-CD4 (CAT#ab183685, abcam), 1:200 dilution<br><br>Flow cytometry antibodies:<br>APC anti-human CD274 (B7-H1, PD-L1) Antibody (CAT#329708, Biolegend), 1:250<br>APC mouse anti-human CD45 (CAT#561863, BD Biosciences), 1:200<br>Anti-human Alexa Fluor 488 dye conjugated antibody (CAT#A-11013, Invitrogen), 1:400<br>APC-Cy7 Rat anti-mouse CD45 (CAT#557659, BD Biosciences), 1:100<br>PerCP Cy5.5 anti-mouse CD3 (CAT# 45-0031-82, eBioscience), 1:100<br>FITC anti-mouse CD8a (CAT#100706 , Biolegend), 1:100<br>BV421 anti-mouse CD69 (CAT#104528, Biolegend), 1:100<br>APC anti-mouse CD107a (CAT#121614, Biolegend), 1:100<br>PE-Cy7 Rat anti-mouse IFN-γ (CAT#557649, Biosciences), 1:50<br><br>In vivo treatment:<br>InVivoMab anti-mouse PD-L1 (B7-H1) (CAT#BE0101, Bioxcell)
Validation	Rabbit anti-PD-L1 (CAT#13684, CST): Specificity/Sensitivity: PD-L1 (E1L3N®) XP® Rabbit mAb recognizes human endogenous levels of total PD-L1 protein. Application: Western blotting, Immunoprecipitation, Immunohistochemistry, IF, Flow Cytometry.<br>Rabbit anti-PD-L1 (CAT#17952-1-AP, Proteintech): PD-L1 Rabbit Polyclonal Ab, Species specificity: human, mouse, rat. Application: WB, IP, IHC, IF, FC, ELISA.<br>Mouse anti-PD-L1 (CAT#29122, CST), Specificity/Sensitivity: PD-L1 (405.9A11) Mouse mAb recognizes human endogenous levels of total PD-L1 protein. Application: Western blotting, Immunohistochemistry.<br>Mouse anti-Trappc4 (CAT#ab57364, abcam): TRAPPC4 Mouse Monoclonal Ab, Species specificity: human. Application: WB<br>Rabbit anti-Trappc4 (CAT#HPA041371, Sigma-Aldrich): species reactivity: rat, mouse, human. Application: immunoblotting, immunofluorescence, immunohistochemistry.<br>Mouse anti-Trappc4 (CAT#H00051399-M01, Abnova): TRAPPC4 monoclonal antibody (M01), clone 2D10, reactivity: human, mouse. Application: ELISA, IF, S-ELISA, WB-Ce, WB-Re<br>Mouse anti-Rab11 (CAT#610656, BD Biosciences): Reactivity: Dog (QC Testing), Human, Mouse, Rat, Chicken (Tested in Development). Application: Western blot (Routinely Tested), Immunohistochemistry (Tested During Development), Immunoprecipitation (Not Recommended)<br>Mouse anti-HA tag (CAT#901513, 16B12 clone, Biolegend): Application: WB - Quality tested, ICC, IP - Verified, FC, Purification - Reported in the literature, not verified in house<br>Anti-GAPDH (CAT#KC-5G5, KANGCHENG Technology): HRP-conjugated Monoclonal Mouse Anti-glyceraldehyde-3-phosphate Dehydrogease (GAPDH). Reactivity: Human, Mouse, Rat, Chicken, Rabbit, Fish<br>Rabbit anti-Giantin (CAT#ab80864, abcam): Rabbit polyclonal to Giantin- Golgi Marker. Suitable for: IHC-P, ICC/IF, WB. Reacts with: Human. Knockout validated.<br>Rabbit anti-EEA1 (CAT#ab2900, abcam): Rabbit polyclonal to EEA1 - Early Endosome Marker. Suitable for: ICC/IF, WB. Reacts with: Mouse, Rat, Dog, Human, Xenopus laevis, Chinese hamster. Knockout validated.

Mouse anti-Lamp1 (CAT#ab25630, abcam): Mouse monoclonal [H4A3]: Mouse monoclonal [H4A3] to LAMP1. Reacts with: Rat, Human, Monkey. Suitable for: ICC/IF, Flow Cyt, IHC-Fr, WB, IHC-P.
Rabbit anti-CD8 (CAT#98941, CST): CD8α (D4W2Z) XP® Rabbit mAb (Mouse Specific). Reactivity: WB, IHC
Rabbit anti-CD4 (CAT#ab183685, abcam): Rabbit monoclonal [EPR19514] to CD4. Suitable for: IP, IHC-P, IHC-Fr, WB. Reacts with: Mouse
APC anti-human CD274 (B7-H1, PD-L1) Antibody (CAT#329708, Biolegend): Mouse Monoclonal antibody, reacted with Human, African Green, Baboon, Cynomolgus, Rhesus, Application: FC - Quality tested.
APC mouse anti-human CD45 (CAT#561863, BD Biosciences): Reactivity: human. Application: Flow cytometry (Routinely Tested)
APC-Cy7 Rat anti-mouse CD45 (CAT#557659, BD Biosciences): Reactivity: mouse (QC Testing). Application: Flow cytometry (Routinely Tested)
PerCP Cy5.5 anti-mouse CD3 (CAT# 45-0031-82, eBioscience): Reactivity: mouse. Application: Flow, IHC, IHC (F) .
FITC anti-mouse CD8a (CAT#100706, Biolegend): Reactivity: mouse. Application: FC - Quality tested.
BV421 anti-mouse CD69 (CAT#104528, Biolegend): Reactivity: mouse. Application: FC - Quality tested.
APC anti-mouse CD107a (CAT#121614, Biolegend): Reactivity: mouse. Application: FC - Quality tested.
PE-Cy7 Rat anti-mouse IFN-γ BD (CAT#557649, Biosciences): Reactivity: mouse (QC Testing). Application: Intracellular staining (flow cytometry) (Routinely Tested)

Eukaryotic cell lines

Policy information about cell lines

Cell line source(s)
Human colorectal cancer cell lines HCT-116 (CCL-247), LoVo (CCL-229) and RKO (CRL-2577) were purchased from ATCC.
Mouse colorectal cancer cell lines MC38 (1101MOU-PUMC000523) was purchased from BMCR.

Authentication
All cell lines were authenticated by providers (STR profiling).

Mycoplasma contamination
All cell lines were tested negative for mycoplasma contamination.

Commonly misidentified lines (See ICLAC register)
None commonly misidentified cell lines were used in the experiments.

Palaeontology and Archaeology

Specimen provenance
Provide provenance information for specimens and describe permits that were obtained for the work (including the name of the issuing authority, the date of issue, and any identifying information). Permits should encompass collection and, where applicable, export.

Specimen deposition
Indicate where the specimens have been deposited to permit free access by other researchers.

Dating methods
If new dates are provided, describe how they were obtained (e.g. collection, storage, sample pretreatment and measurement), where they were obtained (i.e. lab name), the calibration program and the protocol for quality assurance OR state that no new dates are provided.

☐ Tick this box to confirm that the raw and calibrated dates are available in the paper or in Supplementary Information.

Ethics oversight
Identify the organization(s) that approved or provided guidance on the study protocol, OR state that no ethical approval or guidance was required and explain why not.

Note that full information on the approval of the study protocol must also be provided in the manuscript.

Animals and other organisms

Policy information about studies involving animals; ARRIVE guidelines recommended for reporting animal research

Laboratory animals
The Trappc4-null mouse line was constructed based on the knockout (KO)-first strategy in the C57BL/6 background. 8-10-week-old male mice were exposed to a CAC challenge based on an azoxymethane (AOM)/ 3 dextran sulfate sodium (DSS) regimen.
Six-to eight-week-old male C57BL/6 mice were purchased from the Shanghai Model Organism Center (Shanghai, China) and quarantined for one week before inoculation of MC38 cells (mouse colon adenocarcinoma cells). Housing condition: 25خ , suitable humidity (typically 50%), 12-hour dark/light cycle.

Wild animals
This study did not involve wild animals.

Field-collected samples
This study did not involve samples collected from the field.

Ethics oversight
All animal experiments were conducted according to the guidelines approved by Renji Hospital, Shanghai Jiaotong University School of Medicine.

Note that full information on the approval of the study protocol must also be provided in the manuscript.

Human research participants

Policy information about studies involving human research participants

Population characteristics	Clinical characteristics of the patient were shown in Supplementary Table 1 (including tumor size, histopathological grade and TNM stage).
Recruitment	Human tissue specimens were collected from 32 patients with CRC from Renji Hospital affiliated to Shanghai Jiao Tong University School of Medicine (Shanghai, China).
Ethics oversight	The study was conducted with approval from the ethics committee of Renji Hospital, Shanghai Jiao Tong University School of Medicine.

Note that full information on the approval of the study protocol must also be provided in the manuscript.

Clinical data

Policy information about clinical studies

All manuscripts should comply with the ICMJEguidelines for publication of clinical research and a completedCONSORT checklist must be included with all submissions.

Clinical trial registration	*Provide the trial registration number from ClinicalTrials.gov or an equivalent agency.*
Study protocol	*Note where the full trial protocol can be accessed OR if not available, explain why.*
Data collection	*Describe the settings and locales of data collection, noting the time periods of recruitment and data collection.*
Outcomes	*Describe how you pre-defined primary and secondary outcome measures and how you assessed these measures.*

Dual use research of concern

Policy information about dual use research of concern

Hazards

Could the accidental, deliberate or reckless misuse of agents or technologies generated in the work, or the application of information presented in the manuscript, pose a threat to:

No	Yes	
☐	☐	Public health
☐	☐	National security
☐	☐	Crops and/or livestock
☐	☐	Ecosystems
☐	☐	Any other significant area

Experiments of concern

Does the work involve any of these experiments of concern:

No	Yes	
☐	☐	Demonstrate how to render a vaccine ineffective
☐	☐	Confer resistance to therapeutically useful antibiotics or antiviral agents
☐	☐	Enhance the virulence of a pathogen or render a nonpathogen virulent
☐	☐	Increase transmissibility of a pathogen
☐	☐	Alter the host range of a pathogen
☐	☐	Enable evasion of diagnostic/detection modalities
☐	☐	Enable the weaponization of a biological agent or toxin
☐	☐	Any other potentially harmful combination of experiments and agents

ChIP-seq

Data deposition

☐ Confirm that both raw and final processed data have been deposited in a public database such as GEO.

☐ Confirm that you have deposited or provided access to graph files (e.g. BED files) for the called peaks.

Data access links
May remain private before publication.

For "Initial submission" or "Revised version" documents, provide reviewer access links. For your "Final submission" document, provide a link to the deposited data.

Files in database submission

Provide a list of all files available in the database submission.

Genome browser session
(e.g. UCSC)

Provide a link to an anonymized genome browser session for "Initial submission" and "Revised version" documents only, to enable peer review. Write "no longer applicable" for "Final submission" documents.

Methodology

Replicates	*Describe the experimental replicates, specifying number, type and replicate agreement.*
Sequencing depth	*Describe the sequencing depth for each experiment, providing the total number of reads, uniquely mapped reads, length of reads and whether they were paired- or single-end.*
Antibodies	*Describe the antibodies used for the ChIP-seq experiments; as applicable, provide supplier name, catalog number, clone name, and lot number.*
Peak calling parameters	*Specify the command line program and parameters used for read mapping and peak calling, including the ChIP, control and index files used.*
Data quality	*Describe the methods used to ensure data quality in full detail, including how many peaks are at FDR 5% and above 5-fold enrichment.*
Software	*Describe the software used to collect and analyze the ChIP-seq data. For custom code that has been deposited into a community repository, provide accession details.*

Flow Cytometry

Plots

Confirm that:

- [x] The axis labels state the marker and fluorochrome used (e.g. CD4-FITC).
- [x] The axis scales are clearly visible. Include numbers along axes only for bottom left plot of group (a 'group' is an analysis of identical markers).
- [x] All plots are contour plots with outliers or pseudocolor plots.
- [x] A numerical value for number of cells or percentage (with statistics) is provided.

Methodology

Sample preparation	Excised tumors were cut into small pieces and digested with collagenase D (1mg/ml, Roche) and DNase I (0.2mg/ml, Roche) at 37خ for 30 minutes. After digestion, the samples were passed through 70µm cell strainers to make single-cell suspension, followed by staining procedure with fluorescently-labeled antibodies. For cytokine staining, the cells were incubated in culture medium containing Leukocyte Activation Cocktail (BD Biosciences) at 37خ for 4 hours. The cells were stained with anti-CD45 (BD Biosciences, 557659), anti-CD3 (eBioscience, 45-0031-82), anti-CD8 (Biolegend, 100706), anti-CD69 (Biolegend, 104528) and anti-CD107a (Biolegend, 121614). After extracellular staining procedure, the cells were washed and resuspended in Fixation/Permeabilization solution (BD Biosciences), followed by staining with anti-IFN-γ (BD Biosciences, 557649).
Instrument	BD LSRFortessa X20 and BD FACSVerse
Software	Samples were collected by FACSDiva (version 6.2) software and analyzed in FlowJo 10.4.
Cell population abundance	We obtained a purity of at least 95% of sorted populations determined by flow cytometry.
Gating strategy	Forward scatter (FSC) and side scatter (SSC) strategies were used for gating followed by an elimination of the doublets based on the size scatter. The isotype control samples were used to define background.

- [x] Tick this box to confirm that a figure exemplifying the gating strategy is provided in the Supplementary Information.

Magnetic resonance imaging

Experimental design

Design type	*Indicate task or resting state; event-related or block design.*
Design specifications	*Specify the number of blocks, trials or experimental units per session and/or subject, and specify the length of each trial or block (if trials are blocked) and interval between trials.*

Behavioral performance measures	*State number and/or type of variables recorded (e.g. correct button press, response time) and what statistics were used to establish that the subjects were performing the task as expected (e.g. mean, range, and/or standard deviation across subjects).*

Acquisition

Imaging type(s)	*Specify: functional, structural, diffusion, perfusion.*
Field strength	*Specify in Tesla*
Sequence & imaging parameters	*Specify the pulse sequence type (gradient echo, spin echo, etc.), imaging type (EPI, spiral, etc.), field of view, matrix size, slice thickness, orientation and TE/TR/flip angle.*
Area of acquisition	*State whether a whole brain scan was used OR define the area of acquisition, describing how the region was determined.*

Diffusion MRI ☐ Used ☐ Not used

Preprocessing

Preprocessing software	*Provide detail on software version and revision number and on specific parameters (model/functions, brain extraction, segmentation, smoothing kernel size, etc.).*
Normalization	*If data were normalized/standardized, describe the approach(es): specify linear or non-linear and define image types used for transformation OR indicate that data were not normalized and explain rationale for lack of normalization.*
Normalization template	*Describe the template used for normalization/transformation, specifying subject space or group standardized space (e.g. original Talairach, MNI305, ICBM152) OR indicate that the data were not normalized.*
Noise and artifact removal	*Describe your procedure(s) for artifact and structured noise removal, specifying motion parameters, tissue signals and physiological signals (heart rate, respiration).*
Volume censoring	*Define your software and/or method and criteria for volume censoring, and state the extent of such censoring.*

Statistical modeling & inference

Model type and settings	*Specify type (mass univariate, multivariate, RSA, predictive, etc.) and describe essential details of the model at the first and second levels (e.g. fixed, random or mixed effects; drift or auto-correlation).*
Effect(s) tested	*Define precise effect in terms of the task or stimulus conditions instead of psychological concepts and indicate whether ANOVA or factorial designs were used.*

Specify type of analysis: ☐ Whole brain ☐ ROI-based ☐ Both

Statistic type for inference<br>(See Eklund et al. 2016)	*Specify voxel-wise or cluster-wise and report all relevant parameters for cluster-wise methods.*
Correction	*Describe the type of correction and how it is obtained for multiple comparisons (e.g. FWE, FDR, permutation or Monte Carlo).*

Models & analysis

n/a	Involved in the study
☐	☐ Functional and/or effective connectivity
☐	☐ Graph analysis
☐	☐ Multivariate modeling or predictive analysis

Functional and/or effective connectivity	*Report the measures of dependence used and the model details (e.g. Pearson correlation, partial correlation, mutual information).*
Graph analysis	*Report the dependent variable and connectivity measure, specifying weighted graph or binarized graph, subject- or group-level, and the global and/or node summaries used (e.g. clustering coefficient, efficiency, etc.).*
Multivariate modeling and predictive analysis	*Specify independent variables, features extraction and dimension reduction, model, training and evaluation metrics.*
